# On the Finite Complexity of Solutions in a Degenerate System of Quadratic Equations: Exact Formula

**DOI:** 10.3390/e25081112

**Published:** 2023-07-25

**Authors:** Olga Brezhneva, Agnieszka Prusińska, Alexey A. Tret’yakov

**Affiliations:** 1 Department of Mathematics, Miami University, Oxford, OH 45056, USA; brezhnoa@miamioh.edu; 2 Faculty of Exact and Natural Sciences, Siedlce University, ul. Konarskiego 2, 08-110 Siedlce, Poland; tret@uph.edu.pl; 3 Systems Research Institute, Polish Academy of Sciences, ul. Newelska 6, 01-447 Warszawa, Poland

**Keywords:** quadratic programming, singular problems, p-regularity, 2-factor-operator, 65H10, 90C20, 65K05, 90C30

## Abstract

The paper describes an application of the *p*-regularity theory to Quadratic Programming (QP) and nonlinear equations with quadratic mappings. In the first part of the paper, a special structure of the nonlinear equation and a construction of the 2-factor operator are used to obtain an exact formula for a solution to the nonlinear equation. In the second part of the paper, the QP problem is reduced to a system of linear equations using the 2-factor operator. The solution to this system represents a local minimizer of the QP problem along with its corresponding Lagrange multiplier. An explicit formula for the solution of the linear system is provided. Additionally, the paper outlines a procedure for identifying active constraints, which plays a crucial role in constructing the linear system.

## 1. Introduction

Consider the nonlinear equation F(x)=0 with the mapping *F* defined by:

(1) $$F\left(x\right)=B{\left[x\right]}^{2}+Mx+N,$$

where $M\in R^{n\times n}$ is a matrix, $N\in R^{n}$ is a vector, and $B:R^{n}\to R^{n}$ is the map defined for $x\in R^{n}$ by:

(2) $$B{\left[x\right]}^{2}=B\left(x,x\right)={\left(x^{T}B_{1}x,\ldots ,x^{T}B_{n}x\right)}^{T},$$

where $B_{i}$ is an n×n symmetric matrix for i=1,…,n.

We also consider the quadratic programming (QP) problem with inequality constraints:

(3) $$\begin{array}{cc} \underset{x}{\mathrm{minimize}} & f\left(x\right)=\frac{1}{2}x^{T}Qx+c^{T}x\\ \mathrm{subject}\;\mathrm{to} & Ax\leq b \end{array}\;\;\left(\mathrm{QP}\right),$$

where *Q* is an n×n symmetric matrix, *A* is an m×n matrix, $c,x\in R^{n}$, and $b\in R^{m}$.

The paper describes an application of the *p*-regularity theory to nonlinear equations with the mapping *F* introduced in (1) and to the quadratic programming problem (3).

In recent years, there has been growing interest in nonlinear problems, including quadratic and polynomial equations, as well as nonlinear optimization problems, attracting specialists from various disciplines (see, for example, refs. [1,2,3,4] and references therein). Furthermore, it was observed that nonlinear problems are closely related to singular problems, as demonstrated in [5]. In fact, it has been discovered that essentially nonlinear problems and singular problems are locally equivalent. In this work, we aim to provide a theoretical foundation for this claim by introducing several auxiliary concepts as proposed in [5].

**Definition 1.**  Let V be a neighborhood of $x^{*}$ in $R^{n}$, and let $U\subset R^{n}$ be a neighborhood of 0. A mapping $F:V\to R^{n}$, where $F\in C^{2}\left(V\right)$, is considered essentially nonlinear at $x^{*}$ if there exists a perturbation of the form:

$$\tilde{F}\left(x^{*}+x\right)=F\left(x^{*}+x\right)+\omega \left(x\right),\;\mathrm{where}\;∥\omega \left(x\right)∥=o(∥x∥),$$

such that no nondegenerate transformation of coordinates φ(x):U→V, where $\varphi \in C^{1}\left(U\right)$, satisfies $\varphi \left(0\right)=x^{*}$, $\varphi^{\prime }\left(0\right)=I_{n}$, where $I_{n}$ is the identity map in $R^{n}$, and:

$$\tilde{F}\left(\varphi \left(x\right)\right)=\tilde{F}\left(x^{*}\right)+{\tilde{F}}^{\prime }\left(x^{*}\right)x\;\;\;\mathrm{for}\;\mathrm{all}\;x\in U.$$

**Definition 2.**  We say that the mapping F is singular (or degenerate) at $x^{*}$ if it fails to be regular, meaning its derivative is not onto:

ImF′(x*)≠Rn.

The relationship between the notions of essential nonlinearity and singularity is established in Theorem 1, which was derived in [5].

**Theorem 1.**  Let V be a neighborhood of $x^{*}$ in $R^{n}$. Suppose $F:V\to R^{n}$ is $C^{2}$ and that $x^{*}$ is a solution of F(x)=0. Then F is essentially nonlinear at the point $x^{*}$ if and only if F is singular at the point $x^{*}$.

The work presented in [5] primarily focuses on the construction of *p*-regularity and its applications in various areas of mathematics. However, it does not specifically cover quadratic nonlinear equations and quadratic programming problems. The current paper builds upon the foundation of the *p*-regularity theory established in [5] but introduces novel results. The main objective of this paper is to explore the key aspects of nonlinear problems, with a particular emphasis on systems of quadratic equations and quadratic programming problems that may involve singular solutions.

Specifically, we begin by considering the nonlinear equation F(x)=0. One of the *main goals* of the paper is to derive the exact formula for a solution $x^{*}$ of the nonlinear equation F(x)=0 using the special form of the quadratic mapping *F* defined in (1). We demonstrate how to use a construction of a special 2-factor-operator to transform the original problem into a system of linear equations. The construction of the 2-factor-operator combines the mapping *F* with its first derivative $F^{\prime }\left(x\right)$.

In the second part of the paper, we apply a similar approach to the quadratic programming problem (3) in order to derive explicit formulas for the solution $\left(x^{*},\lambda^{*}\right)$, where $x^{*}$ represents a local minimizer of the QP problem and $\lambda^{*}$ is the corresponding Lagrange multiplier. Namely, using the special form of the QP problem and the 2-factor-operator, we reduce the system of optimality conditions for the QP problem to a system of linear equations, with the point $\left(x^{*},\lambda^{*}\right)$ as its solution. The paper also describes a procedure for identifying the active constraints, which plays a vital role in constructing the linear system.

Although there is literature on solutions of degenerate systems of quadratic equations, the approach presented in this paper is novel and distinct from the methods proposed by other authors. This approach can be applied to various problems and areas of mathematics where the problem involves solving a degenerate equation F(x)=0 with a quadratic mapping *F*. Such nonlinear problems can arise in the numerical solutions and analysis of ordinary differential equations, partial differential equations, optimal control problems, algebraic geometry, and other fields. In the second part of the paper, we specifically focus on using the methods developed in the first sections to solve the QP problem (3). The quadratic programming problems have attracted the attention of many researchers and scientists, so there is an extensive body of literature on the topic. Some publications in this area include [6,7,8,9,10,11,12].

**The outline of the paper.** The main contribution and novelty of the paper are in the exact formulas for a solution of a nonlinear equation and of the quadratic programming problems, presented in Section 3 and Section 5, respectively.

In Section 2, we recall the main definitions of the *p*-regularity theory, as presented in [5], including the special case of p=2. Additionally, we introduce the *p*-factor method for solving singular nonlinear equations of the form F(x)=0 and describe various versions of the 2-factor method.

Section 3 presents some of the key results of the paper, focusing on the application of a modified 2-factor method to solve the nonlinear equation F(x)=0 with the mapping *F* defined as $F\left(x\right)=B{\left[x\right]}^{2}+Mx+N,$ where $M\in R^{n\times n}$ is a matrix, $N\in R^{n}$ is a vector, and $B:R^{n}\to R^{n}$ is defined by (2). In this section, we introduce multiple approaches to obtain exact formulas for a solution to the nonlinear equation F(x)=0, demonstrating that the proposed methods converge to a solution $x^{*}$ of the nonlinear equation in just one iteration.

Section 4 focuses on an auxiliary result used in other parts of the paper. We present a theorem that describes the properties of a special mapping μ(x), which enables us to propose a procedure for determining *r* linearly independent vectors $f_{i_{k}}^{\prime }\left(x^{*}\right)$, k=1,…,r, at the solution $x^{*}$ of F(x)=0, without needing to know the exact value of $x^{*}$. This procedure relies on information about the system of vectors $\left\{f_{1}^{\prime }\left(x\right),\ldots ,f_{m}^{\prime }\left(x\right)\right\}$ at some point *x* within a small neighborhood of $x^{*}$.

Section 5 presents other novel results, focusing on deriving exact formulas for a solution of quadratic programming problems. The section is divided into three parts. In Section 5.1, we consider regular quadratic programming problems and propose three approaches to solving the QP problem and obtaining a formula for its solution. These approaches are based on the construction of the 2-factor-operator. Section 5.2 addresses the issue of identifying the active constraints and proposes strategies for numerically determining the set of active constraints $I(x^{*})$. These techniques are then applied in the final part, Section 5.3, to address degenerate QPs. The paper concludes with some closing remarks in Section 6.

Notation. Let $a_{i}$ denote the rows of the m×n matrix *A* in problem (3), and let $b={\left(b_{1},\ldots ,b_{m}\right)}^{T}$, so that $a_{i}^{T}\in R^{n}$ and $b_{i}\in R$ for i=1,…,m.

The *active set* $I(x^{*})$ at any feasible point $x^{*}$ of problem (3) is the set of indices of the active constraints at $x^{*}$, i.e., $I\left(x^{*}\right)=\left\{i=1,\ldots ,m\;|\;a_{i}x^{*}-b_{i}=0\right\}$.

Furthermore, $\mathrm{Ker}\;S=\{x\in R^{n}|Sx=0\}$ denotes the *null-space (kernel)* of a given linear operator $S:R^{n}\to R^{m}$, and ImS={y∈Rm∣y=Sx for some x∈Rn} is its *image space*.

Let $B:R^{n}\times R^{n}\to R^{n}$ be a *bilinear symmetric mapping*. The *2-form associated with B* is the map $B{\left[\cdot \right]}^{2}:R^{n}\to R^{n}$ defined by $B{\left[x\right]}^{2}=B\left(x,x\right)$ for $x\in R^{n}$. We also use the following notation: Im2B={y∈Rn∣B[x]2=y for some x∈Rn} and ${\mathrm{Ker}}^{2}B=\left\{x\in R^{n}|B{\left[x\right]}^{2}=0\right\}$. We denote by $N(x^{*})$ and $N_{\varepsilon }\left(x^{*}\right)$*neighborhoods* of a point $x^{*}$, where $N_{\varepsilon }\left(x^{*}\right)$ is an ε-neighborhood of $x^{*}$, i.e., an open ball of radius ε centered at $x^{*}$.

The notation for the *scalar (dot) product* of vectors *x* and *y* in $R^{n}$, used in the paper, is $x\cdot y=x^{T}y$.

We denote by $\mathrm{span}(a^{1},\ldots ,a^{m})$ the *linear span* of the given vectors $a^{1},\ldots ,a^{m}$. We also denote by d(x,S) the *distance* between a point *x* and a set *S*.

## 2. Elements of the p-Regularity Theory

We begin this section with the main definitions of the *p*-regularity theory, which are given in [5]. The primary focus is on the sufficiently smooth mapping *F* from $R^{n}$ to $R^{n}$, defined as:

(4) $$F\left(x\right)={\left(f_{1}\left(x\right),\ldots ,f_{n}\left(x\right)\right)}^{T},$$

where $f_{i}\left(x\right):R^{n}\to R$ for i=1,…,n. After presenting the general case, we focus on the specific case of p=2. We introduce the definitions of the 2-regular mapping and the 2-factor-operator, which play a central role in the subsequent sections.

### 2.1. The Main Definitions and Constructions of the p-Regularity Theory

Throughout this section, we consider the nonlinear equation:

(5) F(x)=0,

where *F* is defined in Equation (4). Let $x^{*}\in R^{n}$ represent a solution to the nonlinear Equation (5).

The mapping *F* is called *regular* at $x^{*}$ if:

(6) ImF′(x*)=Rn,

or in other words, if:

$$\mathrm{rank}\;F{\;}^{\prime }\left(x^{*}\right)=n,$$

where $F^{\prime }\left(x^{*}\right)$ is the Jacobian matrix of the mapping *F* at $x^{*}$. Conversely, the mapping *F* is called *nonregular* (*irregular, degenerate*) if the regularity condition (6) is not satisfied.

Let the space $R^{n}$ be decomposed into the direct sum:

(7) $$R^{n}=Y_{1}⊕\ldots ⊕Y_{p},$$

where Y1=ImF′(x*), is defined as the closure of the image of the first derivative of *F* evaluated at $x^{*}$, and *p* is chosen as the minimum number for which Equation (7) holds.

The remaining spaces are defined as follows. Let $Z_{1}=R^{n}$, and let $Z_{2}$ be a closed complementary subspace to $Y_{1}$. Let $P_{Z_{2}}:R^{n}\to Z_{2}$ be the projection operator onto $Z_{2}$ along $Y_{1}$. Define $Y_{2}$ as the closed linear span of the image of the quadratic map $P_{Z_{2}}F^{\left(2\right)}\left(x^{*}\right){\left[\cdot \right]}^{2}$. More generally, we define $Y_{i}$ inductively for i=2,⋯,p−1 as:

Yi=spanImPZiF(i)(x*)[·]i⊆Zi,

where $Z_{i}$ is a choice of a complementary subspace for $\left(Y_{1}⊕\ldots ⊕Y_{i-1}\right)$ with respect to *Y*, i=2,⋯,p, and $P_{Z_{i}}:Y\to Z_{i}$ is the projection operator onto $Z_{i}$ along $\left(Y_{1}⊕\ldots ⊕Y_{i-1}\right)$ with respect to *Y*, i=2,⋯,p. Finally, we let $Y_{p}=Z_{p}.$

Define the following mappings:

(8) $$F_{i}\left(x\right):R^{n}\to Y_{i},\;F_{i}\left(x\right)=P_{Y_{i}}F\left(x\right),\;i=1,\;\cdots ,p,$$

where $P_{Y_{i}}:R^{n}\to Y_{i}$ is the projection operator onto $Y_{i}$ along $\left(Y_{1}⊕\ldots ⊕Y_{i-1}⊕Y_{i+1}⊕\ldots ⊕Y_{p}\right)$ with respect to $R^{n}$ for i=1,⋯,p. Then *F* can be represented as $F\left(x\right)=F_{1}\left(x\right)+\ldots +F_{p}\left(x\right)$ or equivalently as $F\left(x\right)=(F_{1}\left(x\right),\ldots ,F_{p}\left(x\right)).$

**Definition 3.**  *The linear operator $\Psi_{p}\left(h\right)\in L\left(R^{n},Y_{1}⊕\ldots ⊕Y_{p}\right)$, where $h\in R^{n}$, h≠0, is defined by:*

$$\Psi_{p}\left(h\right)=F_{1}^{\prime }\left(x^{*}\right)+F_{2}^{\prime \prime }\left(x^{*}\right)\left[h\right]+\ldots +F_{p}^{\left(p\right)}\left(x^{*}\right){\left[h\right]}^{p-1},$$

*and is called the p*-factor operator.

Consider the nonlinear operator $\Psi_{p}{\left[\cdot \right]}^{p}$ defined by:

$$\Psi_{p}{\left[x\right]}^{p}=F_{1}^{\prime }\left(x^{*}\right)\left[x\right]+F_{2}^{\prime \prime }\left(x^{*}\right){\left[x\right]}^{2}+\ldots +F_{p}^{\left(p\right)}\left(x^{*}\right){\left[x\right]}^{p}.$$

Notice that $\Psi_{p}{\left[x\right]}^{p}=\Psi_{p}\left(x\right)\left[x\right]$.

**Definition 4.**  The p-kernel of the operator $\Psi_{p}$ at the point $x^{*}$ is the set $H_{p}\left(x^{*}\right)$ defined by:

$$H_{p}\left(x^{*}\right)={\mathrm{Ker}}^{p}\Psi_{p},$$

*where:*

$${\mathrm{Ker}}^{p}\Psi_{p}=\left\{h\in R^{n}|F_{1}^{\prime }\left(x^{*}\right)\left[h\right]+F_{2}^{\prime \prime }\left(x^{*}\right){\left[h\right]}^{2}+\ldots +F_{p}^{\left(p\right)}\left(x^{*}\right){\left[h\right]}^{p}=0\right\}.$$

Please note that ${\mathrm{Ker}}^{p}\Psi_{p}=\overset{p}{\underset{k=1}{⋂}}{\mathrm{Ker}}^{k}F_{k}^{\left(k\right)}\left(x^{*}\right)$, where:

$${\mathrm{Ker}}^{k}F_{k}^{\left(k\right)}\left(x^{*}\right)=\left\{\xi \in R^{n}|F_{k}^{\left(k\right)}\left(x^{*}\right){\left[\xi \right]}^{k}=0\right\}$$

is the *k*-kernel of $F^{\left(k\right)}\left(\cdot \right){\left[\cdot \right]}^{k}$.

**Definition 5.**  A mapping F is called p-regular at $x^{*}$ along h if ImΨp(h)=Rn.

**Definition 6.**  A mapping F is called p-regular at $x^{*}$ if it is p-regular along all $h\in H_{p}\left(x^{*}\right)\setminus \left\{0\right\}$ or $H_{p}\left(x^{*}\right)=\left\{0\right\}$.

Now, we will focus on the special case of p=2, which we are using in the paper. We denote the image of the Jacobian matrix $F{\;}^{\prime }\left(x^{*}\right)$ by $R_{1}$: R1=ImF′(x*), and the orthogonal complementary subspace of $R_{1}$ in $R^{n}$ by $R_{2}$. Then:

$$R^{n}=R_{1}⊕R_{2}.$$

We also denote an n×n matrix of the orthogonal projection onto $R_{i}$ in $R^{n}$ by $P_{R_{i}}$, i=1,2.

Similarly to Equation (8), we introduce the mappings:

$$F_{i}\left(x\right)=P_{R_{i}}F\left(x\right),\;i=1,2.$$

The *p*-factor operator plays the central role in the *p*-regularity theory. We give the following definition of the *p*-factor-operator for p=2.

**Definition 7.**  We define a 2–factor-operator of the mapping F at $x^{*}$ with respect to some vector $h\in R^{n}$, h≠0, as a linear operator from $R^{n}$ to $R^{n}$, defined by one of the following equations:

(9) $$\Psi_{2}\left(h\right)=F_{1}{\;}^{\prime }\left(x^{*}\right)+F_{2}{\;}^{\prime \prime }\left(x^{*}\right)\left[h\right],$$

(10) $${\bar{\Psi }}_{2}\left(h\right)=F{\;}^{\prime }\left(x^{*}\right)+P_{R_{2}}F{\;}^{\prime \prime }\left(x^{*}\right)\left[h\right],$$

(11) $${\tilde{\Psi }}_{2}\left(h\right)=F{\;}^{\prime }\left(x^{*}\right)+F{\;}^{\prime \prime }\left(x^{*}\right)\left[h\right].$$

Now we are ready to introduce another very important definition of the 2-regularity theory.

**Definition 8.**  The mapping F is called 2-regular at the point $x^{*}$ with respect to the element h if the image of a 2–factor-operator, defined by one of the Equations (9)–(11) is equal to $R^{n}$.

**Definition 9.**  The mapping F is called 2-regular at $x^{*}$ if it is 2-regular at $x^{*}$ with respect to all the elements h from a set that is defined as:

*1.*  $H_{2}\left(x^{*}\right)=\mathrm{Ker}F_{1}^{\prime }\left(x^{*}\right)⋂{\mathrm{Ker}}^{2}F_{2}^{\prime \prime }\left(x^{*}\right)$ for $\Psi_{2}\left(h\right)$ defined by (9);
*2.*  ${\bar{H}}_{2}\left(x^{*}\right)=\mathrm{Ker}F{\;}^{\prime }\left(x^{*}\right)⋂{\mathrm{Ker}}^{2}P_{R_{2}}F{\;}^{\prime \prime }\left(x^{*}\right)$ for ${\bar{\Psi }}_{2}\left(h\right)$ defined by (10);
*3.*  ${\tilde{H}}_{2}\left(x^{*}\right)=\mathrm{Ker}F{\;}^{\prime }\left(x^{*}\right)⋂{\mathrm{Ker}}^{2}F{\;}^{\prime \prime }\left(x^{*}\right)$ for ${\tilde{\Psi }}_{2}\left(h\right)$ defined by (11).

### 2.2. The *p*-Factor-Method for Solving Singular Nonlinear Equations

In this section, we introduce the *p*-factor-method for solving the singular nonlinear equation F(x)=0. Then we consider the special case of p=2 and describe several versions of the 2-factor-method.

Consider Equation (5) in the case when mapping $F^{\prime }\left(x\right)$ is singular at $x^{*}$. In this case, the *p*-factor method is an iterative procedure defined by:

(12) $$\begin{array}{ccc} x^{k+1} & = & x^{k}-{\left(F^{\prime }\left(x^{k}\right)+P_{2}F^{\prime \prime }\left(x^{k}\right)\left[h\right]+\ldots +P_{p}F^{\left(p\right)}\left(x^{k}\right){\left[h\right]}^{p-1}\right)}^{-1}\\ & & \cdot \left(F\left(x^{k}\right)+P_{2}F^{\prime }\left(x^{k}\right)\left[h\right]+\ldots +P_{p}F^{\left(p-1\right)}\left(x^{k}\right){\left[h\right]}^{p-1}\right), \end{array}$$

where k=0,1,…, $P_{i}=P_{Y_{i}}$ for i=1,2,…,p, and vector *h*, ∥h∥=1, is chosen in such a way that the *p*-factor operator $\Psi_{p}$ is nonsingular, which implies that the mapping *F* is *p*-regular at $x^{*}$ along *h*. The following theorem is valid for the *p*-factor-method (12).

**Theorem 2.**  Assume that mapping $F\in C^{p+1}\left(R^{n}\right)$ and there exists vector h, ∥h∥=1, such that the p-factor operator $\Psi_{p}$ is nonsingular. Given a point $x^{0}\in N_{\varepsilon }\left(x^{*}\right)$, where ε>0 is sufficiently small and $N_{\varepsilon }\left(x^{*}\right)$ is a neighborhood of $x^{*}$, the sequence $\left\{x^{k}\right\}$ defined by Equation (12) converges quadratically to the solution $x^{*}$ of (5):

(13) $$∥x^{k+1}-x^{*}∥\leq C∥x^{k}-x^{*}{∥}^{2},\;k=0,1,\ldots ,$$

*where C>0 is an independent constant.*

Now, we are ready to describe several versions of the 2-factor-method.

For solving singular nonlinear Equation (5), the following iterative method, called the *2-factor-method*, was proposed in [13]:

(14) $$x^{k+1}=x^{k}-{\left(F{\;}^{\prime }\left(x^{k}\right)+P_{R_{2}}F{\;}^{\prime \prime }\left(x^{k}\right)\left[h\right]\right)}^{-1}\left(F\left(x^{k}\right)+P_{R_{2}}F{\;}^{\prime }\left(x^{k}\right)\left[h\right]\right),\;\;k=0,1,2,\ldots ,$$

where the vector *h*, ∥h∥=1, is chosen in such a way that matrix $\left(F{\;}^{\prime }\left(x^{*}\right)+P_{R_{2}}F{\;}^{\prime \prime }\left(x^{*}\right)\left[h\right]\right)$ is invertible.

The following theorem states the convergence properties of the 2-factor-method (14).

**Theorem 3.**  Given a mapping $F\in C^{3}\left(R^{n}\right)$, let $x^{*}$ be a solution of Equation (5). Assume that there exists a vector $h\in R^{n}$ such that ∥h∥=1 and F is 2-regular at the point $x^{*}$ with respect to the vector h with the 2-factor-operator ${\bar{\Psi }}_{2}\left(h\right)$ defined by (10).

Then there is a neighborhood $N(x^{*})$ of $x^{*}$ in $R^{n}$ such that for any $x^{0}\in N\left(x^{*}\right)$, the sequence $\left\{x^{k}\right\}$ generated by the 2-factor-method (14) converges to $x^{*}$ and:

(15) $$∥x^{k+1}-x^{*}∥\leq C∥x^{k}-x^{*}{∥}^{2},$$

*where C>0 is some constant.*

**Proof.**  Since $P_{R_{2}}$ is the orthoprojector onto subspace $R_{2}=R_{1}^{⊥}$, then for the mapping:

$$\Phi \left(x\right)=F\left(x\right)+P_{R_{2}}F{\;}^{\prime }\left(x\right)\left[h\right],$$

we have $\Phi (x^{*})=0$. Moreover, because $\Phi^{\prime }\left(x^{*}\right)={\bar{\Psi }}_{2}\left(h\right)$ and the mapping *F* is 2-regular with respect to the vector *h*, by Definition 8 with ${\bar{\Psi }}_{2}\left(h\right)$ defined by (10), we obtain that ImΨ¯2(h)=Rn. Hence, the matrix $\Phi^{\prime }\left(x^{*}\right)$ is invertible.

Therefore, the 2-factor-method given in (14) is an application of Newton’s method to system Φ(x)=0 in a sufficiently small neighborhood of $x^{*}$. Then the statement of the theorem follows from the properties of Newton’s method [14] (Proposition 1.4.1). □

Now, we will introduce a modified version of the 2-factor-method (14). Assume that there exists a vector h≠0 such that $F{\;}^{\prime }\left(x^{*}\right)h=0$ and the matrix $(F{\;}^{\prime }\left(x^{*}\right)$$+F{\;}^{\prime \prime }\left(x^{*}\right)\left[h\right])$ is invertible. Then for solving Equation (5), we can use the following modified 2-factor-method:

(16) $$x^{k+1}=x^{k}-{\left(F{\;}^{\prime }\left(x^{k}\right)+F{\;}^{\prime \prime }\left(x^{k}\right)\left[h\right]\right)}^{-1}\left(F\left(x^{k}\right)+F{\;}^{\prime }\left(x^{k}\right)\left[h\right]\right),\;k=0,1,2,\ldots .$$

The following theorem states the convergence properties of method (16).

**Theorem 4.**  Given a mapping $F\in C^{3}\left(R^{n}\right)$, let $x^{*}$ be a solution of Equation (5). Assume that there exists a vector $h\in R^{n}$ such that ∥h∥=1, $F{\;}^{\prime }\left(x^{*}\right)h=0$, and F is 2-regular at the point $x^{*}$ with respect to the vector h, where the 2-factor-operator ${\tilde{\Psi }}_{2}\left(h\right)$ is defined by (11).

Then there is a neighborhood $N(x^{*})$ of $x^{*}$ in $R^{n}$ such that for any $x^{0}\in N\left(x^{*}\right)$, the sequence $\left\{x^{k}\right\}$ generated by the 2-factor-method (16) converges to $x^{*}$, and relation (15) holds with some C>0.

The proof is similar to one of Theorem 3.

Now we introduce another version of the 2-factor-method.

Assume that the following conditions hold:

(17) $$\begin{array}{c} 1.\;\mathrm{There}\;\mathrm{exists}\;a\;\mathrm{vector}\;h\neq 0\;\mathrm{such}\;\mathrm{that}\;F{\;}^{\prime }\left(x^{*}\right)h=0;\\ 2.\;\mathrm{Matrix}\;(F{\;}^{\prime \prime }\left(x^{*}\right)\left[h\right])\;\mathrm{is}\;\mathrm{invertible}. \end{array}$$

Note that if conditions (17) are satisfied, then to solve Equation (5), we can use the following modified 2-factor-method:

(18) $$x^{k+1}=x^{k}-{\left(F{\;}^{\prime \prime }\left(x^{k}\right)\left[h\right]\right)}^{-1}\left(F{\;}^{\prime }\left(x^{k}\right)\left[h\right]\right),\;k=0,1,2,\ldots .$$

For numerical realization of the 2-factor-method in the form (18), we only have to construct vector *h* satisfying conditions (17). Specifics of some problems allow us to choose vector *h* without any knowledge of the solution $x^{*}$. We discuss the choice of the vector *h* in the following sections of the paper.

The following theorem states the convergence properties of method (18).

**Theorem 5.**  Given a mapping $F\in C^{3}\left(R^{n}\right)$, let $x^{*}$ be a solution of Equation (5). Assume that there exists a vector $h\in R^{n}$ such that ∥h∥=1 and conditions (17) are satisfied.

Then, there is a neighborhood $N(x^{*})$ of $x^{*}$ in $R^{n}$ such that for any $x^{0}\in N\left(x^{*}\right)$, the sequence $\left\{x^{k}\right\}$ generated by the 2-factor-method (18) converges to $x^{*}$ and relation (15) holds with some C>0.

The proof is similar to one of Theorem 3.

## 3. Nonlinear Equations with Quadratic Mappings: the Exact Solution Formula

In this section, we consider the mapping *F* defined by Equation (1) as follows:

$$F\left(x\right)=B{\left[x\right]}^{2}+Mx+N,$$

where $M\in R^{n\times n}$ is a matrix, $N\in R^{n}$ is a vector, and $B:R^{n}\to R^{n}$ is the map defined by (2). The mapping *B* is twice continuously differentiable [15], and its derivatives are given by ${\left(B{\left[x\right]}^{2}\right)}^{\prime }=2B\left[x\right]$ and ${\left(B{\left[x\right]}^{2}\right)}^{\prime \prime }=2B$ for some arbitrary $x\in R^{n}$. Let $x^{*}$ denote a solution of the equation F(x)=0.

We will now illustrate the application of the 2-factor method (18) for solving the nonlinear equation F(x)=0 with the mapping *F* defined by (1). We will present multiple approaches to obtain an exact formula for $x^{*}$, with the first approach being a specific case of the second approach. Additionally, we will show that for the mapping *F*, the method (18) converges to $x^{*}$ in just one iteration.

First approach to obtain an exact formula for the solution $x^{*}$.

For the mapping *F* defined by (1), the assumptions (17) of Theorem 5 can be simplified to the existence of a vector *h* that satisfies the following conditions:

(19) $$\begin{array}{c} \left(1\right)\;(2B\left[x^{*}\right]+M)h=0;\\ (2)\;\mathrm{matrix}\;\;(2B[h])\;\mathrm{is}\;\mathrm{invertible}. \end{array}$$

Under these assumptions (19), for the mapping *F* defined by (1) and a given point $x^{0}$, the first iteration of the 2-factor-method (18) can be written as:

$$x^{1}=x^{0}-{\left(2B[h]\right)}^{-1}\left(\left(2B\left[x^{0}\right]+M\right)\left[h\right]\right),$$

which is equivalent to:

$$\left(2B\left[h\right]\right)\left(x^{1}-x^{0}\right)=-\left(\left(2B\left[x^{0}\right]+M\right)\left[h\right]\right).$$

Using the property $2B\left(h,x^{0}\right)=2B\left(x^{0},h\right)$, the last equation implies a one-step method for calculating $x^{1}$ and, consequently, finding the solution $x^{*}$:

(20) $$x^{*}=-\frac{1}{2}{\left(B\left[h\right]\right)}^{-1}\left(Mh\right),$$

where the vector *h* satisfies conditions (19).

The numerical determination of the vector *h* depends on the specific characteristics of the problem. Alternatively, it can be obtained using the same method as described in the third approach below, which involves transforming the initial system into a system that is completely degenerate at the point $x^{*}$.

Second approach to obtain an exact formula for the solution $x^{*}$.

Now we present an alternative approach for obtaining a formula for the solution $x^{*}$ of the equation F(x)=0 using the same mapping *F* defined by (1). This second approach is applicable to a broader variety of problems compared to the first approach.

Let $P_{1}$ denote the projector onto Y1=span(Im2B), and let $P_{2}$ denote the projector onto $Y_{1}^{⊥}$, which is the orthogonal complementary subspace of $Y_{1}$ in $R^{n}$. We note that $P_{2}\left(B{\left[x^{*}\right]}^{2}\right)=0$ and:

$$4P_{2}B\left(x^{*},x\right)=P_{2}\left(B{\left[x+x^{*}\right]}^{2}-B{\left[x-x^{*}\right]}^{2}\right)=0.$$

Then for the mapping *F* defined in (1),

$$P_{1}F{\;}^{\prime }\left(x^{*}\right)=P_{1}\left(2B\left[x^{*}\right]+M\right)\;\;\mathrm{and}\;\;P_{2}F{\;}^{\prime }\left(x^{*}\right)=P_{2}M.$$

Assume that there exists a vector $h\in R^{n}$ satisfying the conditions:

(21) $$\begin{array}{c} \left(1\right)\;P_{1}\left(2B\left[x^{*}\right]+M\right)h=0;\\ \left(2\right)\;\mathrm{matrix}\;\;(P_{2}M+2P_{1}B\left[h\right])\;\;\mathrm{is}\;\mathrm{invertible}. \end{array}$$

Given the definition of $P_{2}$, it follows that $P_{2}B{\left[x^{*}\right]}^{2}=0$. Substituting this into (1), we obtain $P_{2}\left(Mx^{*}+N\right)=0$. Hence, the point $x^{*}$ satisfies the following identities:

$$\begin{array}{c} P_{2}\left(Mx^{*}+N\right)=0,\\ P_{1}\left(2B\left[x^{*}\right]+M\right)h=0. \end{array}$$

By adding these equations and assuming (21), we obtain the exact formula for the solution $x^{*}$:

(22) $$x^{*}=-{\left(P_{2}M+2P_{1}B\left[h\right]\right)}^{-1}\left(P_{2}N+P_{1}Mh\right).$$

**Remark 1.**  *In the case when $P_{1}=I_{n}$ and, hence, $P_{2}=O_{n\times n}$, assumptions (21) become (19), and Equation (22) reduces to (20).*

**Example 1.**  *Consider mapping $F:R^{2}\to R^{2}$ given by:*

(23) $$F\left(x\right)=\left(\begin{array}{c} x_{1}^{2}-x_{2}^{2}-2x_{1}+1\\ x_{1}x_{2}-x_{2} \end{array}\right).$$

*We can represent the mapping F in the form (1) with:*

$$B=\left(\begin{array}{c} \left(\begin{array}{cc} 1 & 0\\ 0 & -1 \end{array}\right)\\ \left(\begin{array}{cc} 0 & 1/2\\ 1/2 & 0 \end{array}\right) \end{array}\right),\;M=\left(\begin{array}{cc} -2 & 0\\ 0 & -1 \end{array}\right),\;and\;N=\left(\begin{array}{c} 1\\ 0 \end{array}\right).$$

The equation F(x)=0 has a locally unique solution $x^{*}={\left(1,0\right)}^{T}$. In this example, $P_{1}=I_{2}$ and $P_{2}=O_{2\times 2}$. Hence, by Remark 1, we apply Equation (20) with $h={\left(1,0\right)}^{T}$ to obtain:

$$x^{*}=-\frac{1}{2}{\left(Bh\right)}^{-1}\left(Mh\right)=-\frac{1}{2}\left(\begin{array}{cc} 1 & 0\\ 0 & 2 \end{array}\right)\;\left(\begin{array}{c} -2\\ 0 \end{array}\right)=\left(\begin{array}{c} 1\\ 0 \end{array}\right),$$

*as claimed.*

In a numerical implementation, an additional procedure is required to construct the vector *h*. Since the exact point $x^{*}$ is not known in advance, we only assume that a sufficiently small neighborhood of $x^{*}$ is provided to apply the procedure.

Third approach to obtain an exact formula for the solution $x^{*}$.

While the first two approaches rely on knowledge of the element *h*, which is determined by $x^{*}$, the third approach does not require such knowledge. Instead, all we need is for the starting point to belong to a sufficiently small neighborhood $N_{\varepsilon }\left(x^{*}\right)$ of $x^{*}$. Specifically, we have $x^{0}\in N_{\varepsilon }\left(x^{*}\right)$, where ε>0 is sufficiently small.

Suppose that at the point $x^{*}$, the first *r* vectors $\left\{f_{1}^{\prime }\left(x^{*}\right),\ldots ,f_{r}^{\prime }\left(x^{*}\right)\right\}$ are linearly independent, where $f_{i}$ is defined in (4) for i=1,…,r. Assume also that the other vectors $\left\{f_{r+1}^{\prime }\left(x^{*}\right),\ldots ,f_{n}^{\prime }\left(x^{*}\right)\right\}$ are linear combinations of the first *r* vectors. Therefore, there exist coefficients $\alpha_{i}^{j}$ such that:

$$f_{j}^{\prime }\left(x^{*}\right)=\sum_{i=1}^{r}\alpha_{i}^{j}f_{i}^{\prime }\left(x^{*}\right),\;j=r+1,\ldots ,n.$$

Let us introduce the subspace L(x) defined by:

$$L\left(x\right)=\mathrm{span}(f_{1}^{\prime }\left(x\right),\ldots ,f_{r}^{\prime }\left(x\right)).$$

We denote the orthogonal projection on the subspace L(x) as $P_{L(x)}$. Then, there exist coefficients $\alpha_{i}^{j}\left(x\right)$ such that:

$$P_{L(x)}f_{j}^{\prime }\left(x\right)=\sum_{i=1}^{r}\alpha_{i}^{j}\left(x\right)f_{i}^{\prime }\left(x\right),\;j=r+1,\ldots ,n.$$

In addition, introduce the notation:

$${\hat{f}}_{j}^{\prime }\left(x\right)=f_{j}^{\prime }\left(x\right)-P_{L(x)}f_{j}^{\prime }\left(x\right),\;j=r+1,\ldots ,n.$$

Then:

(24) $${\hat{f}}_{j}\left(x\right)=f_{j}\left(x\right)-\sum_{i=1}^{r}\alpha_{i}^{j}\left(x\right)f_{i}\left(x\right),\;j=r+1,\ldots ,n.$$

Notice that $x^{*}$ is also a solution of the equation $\hat{F}\left(x\right)=0$, where $\hat{F}\left(x\right)$ is defined as:

$$\hat{F}\left(x\right)={\left(f_{1}\left(x\right),\ldots ,f_{r}\left(x\right),{\hat{f}}_{r+1}\left(x\right),\ldots ,{\hat{f}}_{n}\left(x\right)\right)}^{T}.$$

The definition of $\hat{F}\left(x\right)$ implies that $\hat{F}$ is 2-regular at the point $x^{*}$. In the case that some of the vectors $f_{k^{\prime }}^{\prime }\left(x^{*}\right)$, $k^{\prime }\in \left\{r+1,\ldots ,n\right\},$ are not zero vectors, transformation (24) can be used to reduce those vectors to zero vectors. This ensures that ${\hat{f}}_{k^{\prime }}^{\prime }\left(x^{*}\right)=0$ for all $k^{\prime }\in \left\{r+1,\ldots ,n\right\}$. Therefore, without loss of generality, we can assume that the mapping F(x) satisfies $f_{j}^{\prime }\left(x^{*}\right)=0$ for j=r+1,…,n. An example of a mapping that satisfies these conditions is:

$$F\left(x\right)=\left(\begin{array}{c} x_{1}+x_{2}\\ x_{1}x_{2} \end{array}\right),$$

where n=2, r=1, j=2, $f_{1}\left(x\right)=x_{1}+x_{2}$, $f_{2}\left(x\right)=x_{1}x_{2}$, and $x^{*}={\left(0,0\right)}^{T}.$

Suppose there exist vectors $\xi_{1}\neq 0,\ldots$, $\xi_{r}\neq 0$, and h≠0, and indices $k_{i}\in \left\{r+1,\ldots ,n\right\}$, i=1,…,r, such that the system:

$$\left\{f_{k_{1}}^{{}^{\prime \prime }}\left(x^{*}\right)\xi_{1},\ldots ,f_{k_{r}}^{{}^{\prime \prime }}\left(x^{*}\right)\xi_{r},f_{r+1}^{{}^{\prime \prime }}\left(x^{*}\right)h,\ldots ,f_{n}^{{}^{\prime \prime }}\left(x^{*}\right)h\right\}$$

is linearly independent.

Then the mapping $\bar{F}\left(x\right)$ defined by:

(25) $$\bar{F}\left(x\right)={\left(f_{k_{1}}^{\prime }\left(x\right)\cdot \xi_{1},\ldots ,f_{k_{r}}^{\prime }\left(x\right)\cdot \xi_{r},f_{r+1}^{\prime }\left(x\right)\cdot h,\ldots ,f_{n}^{\prime }\left(x\right)\cdot h\right)}^{T}$$

has $x^{*}$ as its zero, that is $\bar{F}\left(x^{*}\right)=0$. At the same time, compared to the Jacobian matrix of F(x), the matrix:

$${\bar{F}}^{\prime }\left(x^{*}\right)=\left[\begin{array}{c} {\left(f_{k_{1}}^{\prime \prime }\left(x^{*}\right)\xi_{1}\right)}^{T}\\ \ldots \\ {\left(f_{k_{r}}^{\prime \prime }\left(x^{*}\right)\xi_{r}\right)}^{T}\\ {\left(f_{r+1}^{\prime \prime }\left(x^{*}\right)h\right)}^{T}\\ \ldots \\ {\left(f_{n}^{\prime \prime }\left(x^{*}\right)h\right)}^{T} \end{array}\right]$$

is nonsingular. We can, therefore, consider the method:

(26) $$x^{k+1}=x^{k}-{\left({\bar{F}}^{\prime }\left(x^{k}\right)\right)}^{-1}\bar{F}\left(x^{k}\right),\;k=1,2,\ldots .$$

**Theorem 6.**  Given a mapping $F\in C^{2}\left(R^{n}\right)$, let $x^{*}$ be a solution of Equation (5). Assume that there exist vectors $\xi_{1}\neq 0,\ldots$, $\xi_{r}\neq 0$, and h≠0, such that mapping $\bar{F}\left(x\right)$ defined in (25) is nonsingular at $x^{*}$. Let $x^{0}\in N_{\varepsilon }\left(x^{*}\right)$, where $N_{\varepsilon }\left(x^{*}\right)$ is a neighborhood of $x^{*}$ and ε>0 is sufficiently small.

Then the sequence $\left\{x^{k}\right\}$, k=1,2,… defined by (26) is convergent to the point $x^{*}$ with the quadratic rate of convergence, that is:

$$∥x^{k+1}-x^{*}∥\leq C∥x^{k}-x^{*}{∥}^{2},$$

*where C>0 is an independent constant.*

Using definition of mapping *F* given by Equation (1), mappings $f_{i}$ introduced in (4) will have the following form:

$$f_{i}\left(x\right)=x^{T}B_{i}x+M_{i}^{T}x+N_{i},\;i=1,2,\ldots ,n,$$

where $B_{i}$ is an n×n symmetric matrix, $M_{i}\in R^{n}$, and $N_{i}\in R$, i=1,2,…,n.

Given an initial point $x^{0}$, we use the iterative method (26) to obtain:

$$x^{1}=x^{0}-{\left[\begin{array}{c} {\left(2B_{k_{1}}\xi_{1}\right)}^{T}\\ \vdots \\ {\left(2B_{k_{r}}\xi_{r}\right)}^{T}\\ {\left(2B_{r+1}h\right)}^{T}\\ \vdots \\ {\left(2B_{n}h\right)}^{T} \end{array}\right]}^{-1}\left(\begin{array}{c} {\left(2B_{k_{1}}x^{0}+M_{k_{1}}\right)}^{T}\xi_{1}\\ \vdots \\ {\left(2B_{k_{r}}x^{0}+M_{k_{r}}\right)}^{T}\xi_{r}\\ {\left(2B_{r+1}x^{0}+M_{r+1}\right)}^{T}h\\ \vdots \\ {\left(2B_{n}x^{0}+M_{n}\right)}^{T}h \end{array}\right)=$$

$$={\left[\begin{array}{c} {\left(2B_{k_{1}}\xi_{1}\right)}^{T}\\ \vdots \\ {\left(2B_{k_{r}}\xi_{r}\right)}^{T}\\ {\left(2B_{r+1}h\right)}^{T}\\ \vdots \\ {\left(2B_{n}h\right)}^{T} \end{array}\right]}^{-1}\left(\left(\begin{array}{c} {\left(2B_{k_{1}}\xi_{1}\right)}^{T}x^{0}\\ \vdots \\ {\left(2B_{k_{r}}\xi_{r}\right)}^{T}x^{0}\\ {\left(2B_{r+1}h\right)}^{T}x^{0}\\ \vdots \\ {\left(2B_{n}h\right)}^{T}x^{0} \end{array}\right)-\left(\begin{array}{c} {\left(2B_{k_{1}}x^{0}\right)}^{T}\xi_{1}\\ \vdots \\ {\left(2B_{k_{r}}x^{0}\right)}^{T}\xi_{r}\\ {\left(2B_{r+1}x^{0}\right)}^{T}h\\ \vdots \\ {\left(2B_{n}x^{0}\right)}^{T}h \end{array}\right)-\left(\begin{array}{c} M_{k_{1}}^{T}\xi_{1}\\ \vdots \\ M_{k_{r}}^{T}\xi_{r}\\ M_{r+1}^{T}h\\ \vdots \\ M_{n}^{T}h \end{array}\right)\right).$$

Because matrix $B_{i}$ is symmetric for any index *i*, then for any index *j*, we have:

$${\left(2B_{i}\xi_{j}\right)}^{T}x^{0}=\left(2B_{i}\xi_{j}\right)\cdot x^{0}=\xi_{j}\cdot \left(2B_{i}^{T}x^{0}\right)=\xi_{j}\cdot \left(2B_{i}x^{0}\right)={\left(2B_{i}x^{0}\right)}^{T}\xi_{j}.$$

Therefore,

(27) $$x^{1}=-\frac{1}{2}{\left[\begin{array}{c} {\left(B_{k_{1}}\xi_{1}\right)}^{T}\\ \vdots \\ {\left(B_{k_{r}}\xi_{r}\right)}^{T}\\ {\left(B_{r+1}h\right)}^{T}\\ \vdots \\ {\left(B_{n}h\right)}^{T} \end{array}\right]}^{-1}\left(\begin{array}{c} M_{k_{1}}\cdot \xi_{1}\\ \vdots \\ M_{k_{r}}\cdot \xi_{r}\\ M_{r+1}\cdot h\\ \vdots \\ M_{n}\cdot h \end{array}\right)=x^{*}$$

**Example 1**  *(Continuation). Consider mapping $F:R^{2}\to R^{2}$ defined in (23):*

$$F\left(x\right)=\left(\begin{array}{c} x_{1}^{2}-x_{2}^{2}-2x_{1}+1\\ x_{1}x_{2}-x_{2} \end{array}\right),$$

*where*

$$B_{1}=\left[\begin{array}{cc} 1 & 0\\ 0 & -1 \end{array}\right],\;B_{2}=\left[\begin{array}{cc} 0 & 1/2\\ 1/2 & 0 \end{array}\right],$$

$$M_{1}={\left(-2,0\right)}^{T},\;M_{2}={\left(0,-1\right)}^{T},\;N_{1}=1,\;N_{2}=0.$$

In this example, $x^{*}={\left(1,0\right)}^{T}$ is a solution of F(x)=0 and:

$$f_{1}^{\prime }\left(x^{*}\right)={\left(2x_{1}^{*}-2,2x_{2}^{*}\right)}^{T}={\left(0,0\right)}^{T},\;f_{2}^{\prime }\left(x^{*}\right)={\left(x_{2}^{*},x_{1}^{*}-1\right)}^{T}={\left(0,0\right)}^{T}.$$

*Therefore, mapping $\bar{F}$ defined in (25) takes the form:*

$$\bar{F}\left(x\right)=\left(\begin{array}{c} f_{1}^{\prime }\left(x\right)\cdot h\\ f_{2}^{\prime }\left(x\right)\cdot h \end{array}\right),$$

where h is chosen in such a way that the matrix ${\bar{F}}^{\prime }\left(x^{*}\right)$ is nonsingular, and vectors $\xi_{i}$ are not used. For example, we can take $h={\left(1,0\right)}^{T}$. Then Equation (27) has the form:

(28) $$\begin{array}{ccc} x^{1} & = & -\frac{1}{2}{\left[\begin{array}{c} {\left(B_{1}h\right)}^{T}\\ {\left(B_{2}h\right)}^{T} \end{array}\right]}^{-1}\left(\begin{array}{c} M_{1}\cdot h\\ M_{2}\cdot h \end{array}\right)=-\frac{1}{2}{\left[\begin{array}{cc} 1 & 0\\ 0 & 1/2 \end{array}\right]}^{-1}\left(\begin{array}{c} -2\\ 0 \end{array}\right)\\ \; & = & -\left[\begin{array}{cc} 1/2 & 0\\ 0 & 1 \end{array}\right]\left(\begin{array}{c} -2\\ 0 \end{array}\right)=\left(\begin{array}{c} 1\\ 0 \end{array}\right), \end{array}$$

*which is a solution of F(x)=0 in this example.*

The approaches described above can be modified to derive other methods for solving the equation F(x)=0. For example, using the equation $F^{\prime }{\left(x\right)}^{T}h=0$, where $h\in \mathrm{Ker}\;F^{\prime }{\left(x^{*}\right)}^{T}$, we obtain the following method:

$$x^{k+1}=x^{k}-{\left({\left(F^{\prime }{\left(x^{k}\right)}^{T}\right)}^{\prime }h\right)}^{-1}\left({\left(F^{\prime }\left(x^{k}\right)\right)}^{T}h\right),\;k=0,1,\ldots .$$

The sequence $\left\{x_{k}\right\}$ converges to $x^{*}$ under the assumption that ${\left(F^{\prime }{\left(x^{*}\right)}^{T}\right)}^{\prime }h$ is nonsingular. In this modification, unlike the second approach, we can construct an element *h* without the knowledge of the point $x^{*}$, based on the information at an initial point $x^{0}$.

Applying the modified method to Example 1, we obtain the same formulas and results as shown in Equation (28) above. To implement this approach, it is necessary to determine the vectors $f_{i}^{\prime }\left(x\right)$, i=1,2,…,n, which correspond to linearly independent vectors $f_{i}^{\prime }\left(x^{*}\right),$i∈{1,2,…,n}. This can be achieved using information at a point $x^{0}\in N_{\varepsilon }\left(x^{*}\right)$, where ε>0 is sufficiently small. If the assumption of *p*-regularity is satisfied, the identification of linearly independent vectors is performed using the method described in the next section.

## 4. Procedure for Identifying Zero Elements

The procedure for identifying zero elements could be used to implement the methods described in the previous sections numerically. Let $F\left(x\right):R^{n}\to R^{m}$ be defined as:

(29) $$F\left(x\right)={\left(f_{1}\left(x\right),\ldots ,f_{m}\left(x\right)\right)}^{T}.$$

In this section, we present a theorem that describes the properties of a special mapping μ(x), which allows us to propose the method for determining *r* linear independent vectors $f_{i_{k}}^{\prime }\left(x^{*}\right)$, k=1,…,r, at the solution $x^{*}$ of F(x)=0. This procedure is based on the information about the system of vectors $\left\{f_{1}^{\prime }\left(x\right),\ldots ,f_{m}^{\prime }\left(x\right)\right\}$ at some point *x* in a small neighborhood of $x^{*}$. As a result, we can define the mapping $\hat{F}\left(x\right)$ with the first *r* components $f_{i_{k}}\left(x\right)$, k=1,…,r, corresponding to the linearly independent vectors $f_{i_{k}}^{\prime }\left(x^{*}\right)$, k=1,…,r.

Let $F\in C^{3}\left(R^{n}\right)$ be 2-regular at the point $x^{*}$. For some $x\in N_{\varepsilon }\left(x^{*}\right)$, where ε is sufficiently small, we define the following mappings:

$$\rho \left(x\right)=\min_{i=1,\ldots ,m}d\left(f_{i}^{\prime }\left(x\right),\mathrm{span}\left(f_{1}^{\prime }\left(x\right),\ldots ,f_{i-1}^{\prime }\left(x\right),f_{i+1}^{\prime }\left(x\right),\ldots ,f_{m}^{\prime }\left(x\right)\right)\right)$$

and:

(30) $$\mu \left(x\right)=\max\left\{{∥F\left(x\right)∥}^{\frac{1}{2}},\rho \left(x\right)\right\},$$

where d(x,S) denotes the distance between an element *x* and the set *S*. Note that if $F\left(x\right)=f_{1}\left(x\right)$, then $\rho \left(x\right)=∥f_{1}^{\prime }\left(x\right)∥$.

The mapping μ(x) is used to determine the maximum number *r* of linearly independent vectors in the system $\left\{f_{1}^{\prime }\left(x^{*}\right),\ldots ,f_{m}^{\prime }\left(x^{*}\right)\right\}$ using a special procedure that relies on the information about the mapping F(x) at the point $x\in N_{\varepsilon }\left(x^{*}\right)$. The properties of the mapping μ(x) are stated in the following theorem, and the proof can be found in [16].

**Theorem 7**  (Minorant theorem). Let $F\in C^{3}\left(R^{n}\right)$ be 2-regular at the point $x^{*}$, and $F(x^{*})=0$. Then there exist constants ε>0, $C^{\prime }>0$, and $C^{\prime \prime }>0$ such that the following inequality holds for any $x\in N_{\varepsilon }\left(x^{*}\right)$:

$$C^{\prime }∥x-x^{*}∥\leq \mu \left(x\right)\leq C^{\prime \prime }{∥x-x^{*}∥}^{\frac{1}{2}},$$

*where function μ(x) is defined in (30).*

In addition to the properties of the mapping μ(x) given in Theorem 7, we also need the following lemma (for the proof, see [16]).

**Lemma 1.**  *For the non-negative mappings g(x) and μ(x), let the following inequalities hold:*

$$|g\left(x_{1}\right)-g\left(x_{2}\right)|\leq L∥x_{1}-x_{2}∥\;\;\mathrm{for}\;\mathrm{all}\;x_{1},x_{2}\in N_{\sigma }\left(x^{*}\right),$$

$$C^{\prime }∥x-x^{*}∥\leq \mu \left(x\right)\leq C^{\prime \prime }{∥x-x^{*}∥}^{\frac{1}{p}}\;\;\mathrm{for}\;\mathrm{all}\;x\in N_{\sigma }\left(x^{*}\right),$$

*where L,$C^{\prime }$, $C^{\prime \prime }$, and σ are positive constants, with $C^{\prime \prime }\geq C^{\prime }$ and p≥2.*

*Then, there exists a sufficiently small ε>0 such that one of the following conditions holds:*

*1.*  If $g\left(x\right)\leq \sqrt{\mu (x)}$ for all $x\in N_{\varepsilon }\left(x^{*}\right)$, then $g(x^{*})=0.$
*2.*  If $g\left(x\right)>\sqrt{\mu (x)}$ for all $x\in N_{\varepsilon }\left(x^{*}\right)$, then $g(x^{*})\neq 0.$

**Remark 2.**  Based on the assumptions of Lemma 1, there exists a sufficiently small ε>0 such that if the inequality $g\left(\bar{x}\right)\leq \sqrt{\mu (\bar{x})}$ is satisfied for any $\bar{x}\in N_{\varepsilon }\left(x^{*}\right)$, then the inequality $g\left(x\right)\leq \sqrt{\mu (x)}$ is satisfied for all $x\in N_{\varepsilon }\left(x^{*}\right)$, and hence $g(x^{*})=0$.

Similarly, if the inequality $g\left(\bar{x}\right)>\sqrt{\mu (\bar{x})}$ is satisfied for any $\bar{x}\in N_{\varepsilon }\left(x^{*}\right)$, then the inequality $g\left(x\right)>\sqrt{\mu (x)}$ is satisfied for all $x\in N_{\varepsilon }\left(x^{*}\right)$, and hence $g(x^{*})\neq 0$.

Now we are ready to introduce an iterative method that determines indices 1,…,r corresponding to the linearly independent vectors $f_{i}^{\prime }\left(x^{*}\right)$, i=1,…,r.

Method for determining linearly independent gradients at $x^{*}$ (identifying zero elements).

Using Lemma 1 and Remark 2, for a sufficiently small ε>0, $x\in N_{\varepsilon }\left(x^{*}\right)$, and i=1,…,m, consider two possible cases:

**Case 1.** $g\left(x\right)=∥f_{i}^{\prime }\left(x\right)∥\leq \sqrt{\mu (x)}$.

**Case 2.** $g\left(x\right)=∥f_{i}^{\prime }\left(x\right)∥>\sqrt{\mu (x)}$.

In Case 1, according to Remark 2, it follows that $f_{i}^{\prime }\left(x^{*}\right)=0$, whereas in Case 2, we have $f_{i}^{\prime }\left(x^{*}\right)\neq 0$.

**In addition to the properties of the mapping**

Let $F\left(x\right):R^{n}\to R^{m}$ be defined by (29) and $x^{*}$ be a solution of F(x)=0. Let *x* be in $N_{\varepsilon }\left(x^{*}\right)$, where ε is sufficiently small. Define function μ(x) using Equation (30).

**Step 1.** Identify the smallest index $i_{1}$ in the set $S_{1}=\left\{1,\ldots ,m\right\}$ such that $∥f_{i_{1}}^{\prime }\left(x\right)∥>\sqrt{\mu (x)}$. According to Case 2 above, this implies that $f_{i_{1}}^{\prime }\left(x^{*}\right)\neq 0$.
**Step 2.** Use Step 1 to identify if the set $S_{2}=\left\{1,\ldots ,m\right\}\setminus \left\{i_{1}\right\}$ has at least one index *j* such that $f_{j}^{\prime }\left(x^{*}\right)\neq 0$. If it does not, the method is finished. Otherwise, identify the next smallest index $i_{2}$ in the set $S_{1}$ such that the following condition is satisfied:
  $$d\left(f_{i_{2}}^{\prime }\left(x\right),\mathrm{span}\left(f_{i_{1}}^{\prime }\left(x\right)\right)\right)>\sqrt{\mu (x)}.$$

According to Case 2 above, it means that the vectors $f_{i_{1}}^{\prime }\left(x^{*}\right)$ and $f_{i_{2}}^{\prime }\left(x^{*}\right)$ are linearly independent.

**Step *k*.** By this step, we have identified k−1 linearly independent vectors $f_{i_{1}}^{\prime }\left(x^{*}\right)$, *…*, $f_{i_{k-1}}^{\prime }\left(x^{*}\right)$, where k=3,4,…,m. Use Step 1 to identify if the set $S_{k}=\left\{1,\ldots ,m\right\}\setminus \left\{i_{1},\ldots ,i_{k-1}\right\}$ has at least one index *j* such that $f_{j}^{\prime }\left(x^{*}\right)\neq 0$. If it does not, the method is finished. Otherwise, identify the next smallest index $i_{k}\in \left\{1,\ldots ,m\right\}\setminus \left\{i_{1},\ldots ,i_{k-1}\right\}$ such that the following condition is satisfied:

$$d\left(f_{i_{k}}^{\prime }\left(x\right),\mathrm{span}\left(f_{i_{1}}^{\prime }\left(x\right),\ldots ,f_{i_{k-1}}^{\prime }\left(x\right)\right)\right)>\sqrt{\mu (x)}.$$

The inequality implies that the vectors $f_{i_{1}}^{\prime }\left(x^{*}\right)$, …,$f_{i_{k}}^{\prime }\left(x^{*}\right)$ are linearly independent.

Repeat Step *k* until the method is finished.

Without loss of generality, assume that the first *r* vectors $\left\{f_{1}^{\prime }\left(x^{*}\right),\ldots ,f_{r}^{\prime }\left(x^{*}\right)\right\}$ are linearly independent and define mapping $\hat{F}\left(x\right)$ as:

(31) $$\hat{F}\left(x\right)=\left(\begin{array}{c} f_{1}\left(x\right)\\ \vdots \\ f_{r}\left(x\right)\\ {\hat{f}}_{r+1}\left(x\right)\\ \vdots \\ {\hat{f}}_{m}\left(x\right) \end{array}\right),$$

where vectors ${\hat{f}}_{k}\left(x\right)$ are defined in such a way that:

$${\hat{f}}_{k}^{\prime }\left(x^{*}\right)=0,\;k=r+1,\ldots ,m.$$

Namely, let:

$$f_{k}^{\prime }\left(x^{*}\right)=\sum_{i=1}^{r}\alpha_{i}^{k}\left(x^{*}\right)f_{i}^{\prime }\left(x^{*}\right),\;k=r+1,\ldots ,m,$$

be a linear combination of the vectors $f_{1}^{\prime }\left(x^{*}\right)$, *…*, $f_{r}^{\prime }\left(x^{*}\right).$ Coefficients $\alpha^{k}\left(x\right)$ are determined by solving the following system of equations:

$$\left(f_{k}^{\prime }\left(x\right)-\sum_{i=1}^{r}\alpha_{i}^{k}\left(x\right)f_{i}^{\prime }\left(x\right)\right)\cdot f_{j}^{\prime }\left(x\right)=0,\;j=1,2,\ldots ,r,\;k=r+1,\ldots ,m.$$

In addition, define B(x) to be a nonsingular matrix of the form:

$$B\left(x\right)=\left(\begin{array}{cccccc} 1 & \cdots & 0 & 0 & \cdots & 0\\ \vdots & \ddots & \vdots & \vdots & \ddots & \vdots \\ 0 & \cdots & 1 & 0 & \cdots & 0\\ -\alpha_{1}^{r+1}\left(x\right) & \cdots & -\alpha_{r}^{r+1}\left(x\right) & 1 & \cdots & 0\\ \vdots & \ddots & \vdots & \vdots & \ddots & \vdots \\ -\alpha_{1}^{m}\left(x\right) & \cdots & -\alpha_{r}^{m} & 0 & \cdots & 1 \end{array}\right).$$

Let:

$$\alpha^{k}\left(x\right)=\left(\alpha_{1}^{k}\left(x\right),\ldots ,\alpha_{r}^{k}\left(x\right)\right),\;k=r+1,\ldots ,m.$$

Define the following vectors:

$${\hat{f}}_{k}\left(x\right)=f_{k}\left(x\right),\;k=1,\ldots ,r,$$

$${\hat{f}}_{k}\left(x\right)=f_{k}\left(x\right)-\sum_{i=1}^{r}\alpha_{i}^{k}\left(x\right)f_{i}\left(x\right),\;k=r+1,\ldots ,m.$$

These vectors allow us to transform the mapping F(x) to $\hat{F}\left(x\right)=B\left(x\right)\cdot F\left(x\right)$, where ${\hat{f}}_{1}^{\prime }\left(x^{*}\right)\neq 0,$…,${\hat{f}}_{r}^{\prime }\left(x^{*}\right)\neq 0,$ and ${\hat{f}}_{r+1}^{\prime }\left(x^{*}\right)=0,$…,${\hat{f}}_{m}^{\prime }\left(x^{*}\right)=0.$ The purpose of this transformation is to simplify the structure of the projection operators.

We present a simple example to illustrate an application of the proposed method.

**Example 2.**  *Let $F:R^{2}\to R^{2}$, $F\left(x\right)={\left(f_{1}\left(x\right),f_{2}\left(x\right)\right)}^{T}$, where:*

$$f_{1}\left(x\right)=x_{1}+x_{2},\;f_{2}\left(x\right)=x_{1}x_{2}.$$

Then $x^{*}={\left(0,0\right)}^{T}$ is a solution of F(x)=0. Take $\varepsilon =\frac{1}{\sqrt{2}}$ and consider $\bar{x}={\left(\frac{1}{2},\frac{1}{2}\right)}^{T}\in N_{\varepsilon }\left(x^{*}\right)$. The Jacobian matrix of F is:

$$F^{\prime }\left(x\right)=\left(\begin{array}{cc} 1 & 1\\ x_{2} & x_{1} \end{array}\right),$$

$$f_{1}^{\prime }\left(x^{*}\right)=\left(\begin{array}{c} 1\\ 1 \end{array}\right),\;and\;f_{2}^{\prime }\left(x^{*}\right)=\left(\begin{array}{c} 0\\ 0 \end{array}\right).$$

It is easy to see that vectors $f_{1}^{\prime }\left(x^{*}\right)$ and $f_{2}^{\prime }\left(x^{*}\right)$ are linearly dependent. We can check this by applying the method introduced above.

*By using Equation (30), we define function $\mu \left(x\right)=\max\left\{{∥F\left(x\right)∥}^{\frac{1}{2}},\rho \left(x\right)\right\}$, where:*

$$\rho \left(x\right)=\sin\alpha ∥f_{2}^{\prime }\left(x\right)∥,$$

*and α is the angle between vectors $f_{1}^{\prime }\left(x\right)$ and $f_{2}^{\prime }\left(x\right)$. Note that:*

$$\cos\alpha =\frac{f_{1}^{\prime }\left(x\right)\cdot f_{2}^{\prime }\left(x\right)}{∥f_{1}^{\prime }\left(x\right)∥∥f_{2}^{\prime }\left(x\right)∥},$$

*and hence:*

$$\rho \left(x\right)=\sqrt{\frac{∥f_{1}^{\prime }{\left(x\right)∥}^{2}{∥f_{2}^{\prime }\left(x\right)∥}^{2}-{\left(f_{1}^{\prime }\left(x\right)\cdot f_{2}^{\prime }\left(x\right)\right)}^{2}}{∥f_{1}^{\prime }{\left(x\right)∥}^{2}{∥f_{2}^{\prime }\left(x\right)∥}^{2}}}∥f_{2}^{\prime }\left(x\right)∥=\frac{|x_{1}-x_{2}|}{\sqrt{2}}.$$

*Using $\bar{x}={\left(\frac{1}{2},\frac{1}{2}\right)}^{T}$, we obtain:*

$$\rho \left(\bar{x}\right)=0,\;{∥F\left(\bar{x}\right)∥}^{\frac{1}{2}}=\sqrt[{4}]{1^{2}+{\left(\frac{1}{4}\right)}^{2}}=\sqrt[{4}]{\frac{17}{16}},\;\;\mathrm{and}\;\mathrm{so}\;\;\mu \left(\bar{x}\right)=\sqrt[{4}]{\frac{17}{16}}.$$

*We are ready to apply the method described above.*

*In Step 1, we obtain $∥f_{1}^{\prime }\left(\bar{x}\right)∥>\sqrt{\mu (\bar{x})}$ because:*

$$\sqrt{2}>\sqrt[{8}]{\frac{17}{16}}.$$

Hence, vector $f_{1}^{\prime }\left(x^{*}\right)\neq 0$ and $i_{1}=1$.

*Then in Step 1 of the method with vector $f_{2}^{\prime }\left(x\right)$, we also verify whether the following inequality holds:*

$$∥f_{2}^{\prime }\left(\bar{x}\right)∥>\mu {\left(\bar{x}\right)}^{\frac{1}{2}}\;or\;\sqrt{{\bar{x}}_{2}^{2}+{\bar{x}}_{1}^{2}}>\mu {\left(\bar{x}\right)}^{\frac{1}{2}}.$$

*Using point $\bar{x}={\left(\frac{1}{2},\frac{1}{2}\right)}^{T}$, we obtain:*

$$\sqrt{\frac{1}{2}}<\sqrt[{8}]{\frac{17}{16}}.$$

Therefore, we conclude that $f_{2}^{\prime }\left(x^{*}\right)=0$.

*Thus, in this example, the mapping $\hat{F}\left(x\right)$ defined in (31) has the form $\hat{F}\left(x\right)={\left(f_{1},{\hat{f}}_{2}\right)}^{T}$, where $f_{1}\left(x\right)=x_{1}+x_{2}$ and ${\hat{f}}_{2}=x_{1}x_{2}$.*

## 5. Quadratic Programming Problems

In this section, we consider the quadratic programming (QP) problem (3):

$$\begin{array}{cc} \underset{x}{\mathrm{minimize}} & f\left(x\right)=\frac{1}{2}x^{T}Qx+c^{T}x\\ \mathrm{subject}\;\mathrm{to} & Ax\leq b \end{array}\;\;\left(\mathrm{QP}\right),$$

where *Q* is an n×n symmetric matrix, *A* is an m×n matrix, $c,x\in R^{n}$, and $b\in R^{m}$. The Lagrangian for problem (3) is defined by:

(32) $$L\left(x,\lambda \right)=\frac{1}{2}x^{T}Qx+c^{T}x+\sum_{i=1}^{m}\lambda_{i}\left(a_{i}x-b_{i}\right),$$

where $\lambda =(\lambda_{1},\ldots ,\lambda_{m})$ is the vector of Lagrange multipliers and $a_{i}$ is the *i*th row of the matrix *A*. The Karush-Kuhn-Tucker (KKT) conditions [17] are satisfied at $x^{*}$ with some $\lambda^{*}\in R^{m}$ if:

(33) $$\begin{array}{cc} Qx^{*}+c+\sum_{i=1}^{m}\lambda_{i}^{*}a_{i}^{T}=0, & \\ \lambda_{i}^{*}\left(a_{i}x^{*}-b_{i}\right)=0,\;\lambda_{i}^{*}\geq 0,\;a_{i}x^{*}\leq b_{i}, & \;\mathrm{for}\;\;\mathrm{all}\;\;i=1,\ldots ,m. \end{array}$$

The point $x^{*}$ at which relations (33) are satisfied is called a *stationary point* or a *KKT point*. Observe that $\left(x^{*},\lambda^{*}\right)$ is a solution of the following system:

(34) $$\Phi \left(x,\lambda \right)=\left(\begin{array}{c} Qx+c+A^{T}\lambda \\ \Lambda (Ax-b) \end{array}\right)=0,\;\Lambda =\mathrm{diag}{\left(\lambda_{i}\right)}_{i=1,\ldots ,m},\;\lambda_{i}^{*}\geq 0,\;Ax^{*}\leq b.$$

We denote by $I(x^{*})$ the *set of indices of the active constraints* at $x^{*}$:

$$I\left(x^{*}\right)=\left\{i=1,\ldots ,m|a_{i}x^{*}=b_{i}\right\}.$$

The following constraint qualification is used in the paper.

**Definition 10**  (Linear independence constraint qualification). The linear independence constraint qualification (LICQ) holds at a feasible point $x^{*}$ if the row-vectors $a_{j}$, $j\in I(x^{*})$, corresponding to the active at $x^{*}$ constraints, are linearly independent.

The modified second-order sufficient conditions (MSOSC) state that there exist a Lagrange multiplier vector $\lambda^{*}$ and a scalar ν>0 such that:

(35) $$\omega^{T}\nabla^{2}L_{x\;x}\left(x^{*},\lambda^{*}\right)\omega \geq \nu {∥\omega ∥}^{2}$$

for all ω satisfying:

$$a_{i}\omega =0\;\forall i\in I\left(x^{*}\right).$$

We divide the presentation in this section into three parts. We start by considering regular QP problems in Section 5.1. Then, in Section 5.2, we discuss the issue of identifying the active constraints and propose numerical strategies for determining the set $I(x^{*})$. We apply these techniques to degenerate QP problems in Section 5.3.

### 5.1. Regular Quadratic Programming

In this section, we consider regular quadratic programming (QP) problem (3). In other words, we assume that the Linear Independence Constraint Qualification (LICQ) and the Mangasarian-Fromovitz Constraint Qualification (MFCQ) conditions (35) hold. Recall that *A* is an m×n matrix of coefficients representing the constraints Ax≤b in problem (3). Without loss of generality, assume that the first *p* constraints are active at $x^{*}$, so that:

$$I\left(x^{*}\right)=\left\{1,\ldots ,p\right\}.$$

Then we can rewrite the matrix *A* in the following form:

(36) $$A=\left[\begin{array}{c} A_{A}\\ A_{N} \end{array}\right],$$

where $A_{A}$ is a p×n matrix of coefficients corresponding to the active constraints at $x^{*}$, and $A_{N}$ is an (m−p)×n matrix of coefficients corresponding to the nonactive constraints at $x^{*}$. It is important to note that we do not have prior knowledge of the set $I(x^{*})$. We will discuss possible numerical realizations to approximate the set of active constraints in Section 5.2. Additionally, we introduce the following notation associated with the active constraints at the point $x^{*}$:

$$b_{A}={\left(b_{1},\ldots ,b_{p}\right)}^{T},\;\lambda_{A}={\left(\lambda_{1},\ldots ,\lambda_{p}\right)}^{T}.$$

Similarly,

$$b_{N}={\left(b_{p+1},\ldots ,b_{m}\right)}^{T},\;\lambda_{N}={\left(\lambda_{p+1},\ldots ,\lambda_{m}\right)}^{T}.$$

In the following subsections, we will introduce three approaches to solving the QP problem (3) and provide formulas for the solution.

#### 5.1.1. First Approach to Solving the QP Problem

In this subsection, we present an approach to solving the QP problem and obtaining a formula for its solution. This approach is based on the construction of the 2-factor-operator. For our consideration below, we need the following lemma.

**Lemma 2.**  *Let V be an n×n matrix, G be a p×n matrix, such that the columns of $G^{T}$ are linearly independent, L be an n×l matrix, $G_{N}=diag\;{\left(g_{i}\right)}_{i=1}^{l}$ be a diagonal full rank matrix, and:*

(37) (Vx,x)>0 for all x∈KerG∖{0}.

*Then matrix *Γ* defined by:*

(38) $$\Gamma =\left(\begin{array}{ccc} V & G^{T} & L\\ G & O_{p\times p} & O_{p\times l}\\ O_{l\times n} & O_{l\times p} & G_{N} \end{array}\right)$$

*is nonsingular.*

**Proof.**  To prove the lemma, we must prove that the matrix Γ defined by (38) has zero nullspace. Consider the following system that defines the nullspace of Γ in the form of a vector v=(x,y,z), where $x\in R^{n}$, $y\in R^{p}$, and $z\in R^{l}$:

(39) $$\begin{array}{c} Vx+G^{T}y+Lz=0\\ Gx=0\\ G_{N}z=0. \end{array}$$

Since $G_{N}$ is a full-rank diagonal matrix, the third equation in the system (39) implies that z=0. Then, using the first equation, we obtain:

$$\begin{array}{cc} 0 & =\left(Vx\right)\cdot x+\left(G^{T}y\right)\cdot x\\ \; & =(Vx)\cdot x+y\cdot (Gx)\\ \; & =(Vx)\cdot x. \end{array}$$

Consequently, x=0; otherwise, (Vx)·x=0, which contradicts the assumption (37) of the lemma. Therefore, the first equation in (39) reduces to $G^{T}y=0$, and since the columns of $G^{T}$ are linearly independent, we obtain y=0. Thus, the matrix Γ (38) has a zero nullspace, (x,y,z)=(0,0,0), and therefore, Γ is nonsingular. This concludes the proof of the lemma. □

Let $x\in R^{n}$, $\lambda \in R^{m},$ and mapping Φ be defined in (34), so that $\Phi \left(x,\lambda \right)=\left(\begin{array}{c} Qx+c+A^{T}\lambda \\ \Lambda (Ax-b) \end{array}\right)$. Introduce mappings $P_{1}$ and $P_{2}$ as:

$$P_{1}=\left[\begin{array}{cc} I_{n\times n} & O_{n\times m}\\ O_{m\times n} & O_{m\times m} \end{array}\right],\;P_{2}=\left[\begin{array}{cc} O_{n\times n} & O_{n\times m}\\ O_{m\times n} & I_{m\times m} \end{array}\right].$$

Recall that matrix $A_{A}$ is defined in (36), and introduce vector $\bar{h}\in R^{n+m}$ such that:

$$\bar{h}={\left(h^{1},h^{2}\right)}^{T},\;h^{1}\in R^{n},\;h^{2}\in R^{m},$$

where $A_{A}h^{1}=0$, $h^{1}\neq 0$, h2=((1⋯1︸p,(0⋯0︸m−p).

Define mapping Ψ as:

(40) $$\Psi \left(x,\lambda \right)=P_{1}\Phi \left(x,\lambda \right)+P_{2}\Phi^{\prime }\left(x,\lambda \right)\bar{h}.$$

Recall that $a_{i}$ is the *i*th row of the matrix *A* and $b={\left(b_{1},\ldots ,b_{m}\right)}^{T}$. Then:

$$\Phi^{\prime }\left(x,\lambda \right)=\left(\begin{array}{cc} Q & A^{T}\\ \Lambda A & S \end{array}\right),\;S=\left(\begin{array}{ccc} a_{1}x-b_{1} & 0 & 0\\ \; & \ddots & \\ 0 & 0 & a_{m}x-b_{m} \end{array}\right),$$

and mapping Ψ defined in (40) can be rewritten as:

$$\Psi \left(x,\lambda \right)=\left(\begin{array}{c} Qx+c+A^{T}\lambda \\ \Lambda Ah^{1}+Sh^{2} \end{array}\right).$$

Introduce matrix $\Lambda_{N}=\mathrm{diag}{\left(\lambda_{i}\right)}_{i=p+1,\ldots ,m}$. Then, taking into account the definition of $h^{1}$ and $h^{2}$, we obtain:

$$\Psi \left(x,\lambda \right)=\left(\begin{array}{c} Qx+c+A^{T}\lambda \\ A_{A}x-b_{A}\\ \Lambda_{N}A_{N}h^{1} \end{array}\right).$$

Observe that if $\left(x^{*},\lambda^{*}\right)$ is a solution of (34), it is also a solution of Ψ(x,λ)=0, or, equivalently,

(41) $$\Psi \left(x,\lambda \right)=\left(\begin{array}{c} Qx+c+A^{T}\lambda \\ A_{A}x-b_{A}\\ \Lambda_{N}A_{N}h^{1} \end{array}\right)=0.$$

To obtain the formula for the solution $\left(x^{*},\lambda^{*}\right)$, we rewrite the system (41) as:

$$\left(\begin{array}{ccc} Q & A_{A}^{T} & A_{N}^{T}\\ A_{A} & O & O\\ O_{(m-p)\times n} & O & K \end{array}\right)\;\left(\begin{array}{c} x\\ \lambda_{A}\\ \lambda_{N} \end{array}\right)=\left(\begin{array}{c} -c\\ b_{A}\\ O_{m-p} \end{array}\right),\;K=\left(\begin{array}{ccc} a_{p+1}h^{1} & 0 & 0\\ \; & \ddots & \\ 0 & 0 & a_{m}h^{1} \end{array}\right).$$

Assuming that LICQ and MSOSC hold and apply Lemma 2, we obtain that the matrix:

$$\left(\begin{array}{ccc} Q & A_{A}^{T} & A_{N}^{T}\\ A_{A} & O & O\\ O_{(m-p)\times n} & O & K \end{array}\right)$$

is invertible and obtain the formula for $\left(x^{*},\lambda^{*}\right)$:

(42) $$\left(\begin{array}{c} x^{*}\\ \lambda_{A}^{*}\\ \lambda_{N}^{*} \end{array}\right)={\left(\begin{array}{ccc} Q & A_{A}^{T} & A_{N}^{T}\\ A_{A} & O & O\\ O_{(m-p)\times n} & O & K \end{array}\right)}^{-1}\;\left(\begin{array}{c} -c\\ b_{A}\\ O_{m-p} \end{array}\right).$$

#### 5.1.2. Second Approach to Solving the QP Problem

Assume that we can estimate the set $I(x^{*})$, which is in our notation $I\left(x^{*}\right)=\left\{1,2,\ldots ,p\right\}$. Taking into account that $\lambda_{p+1}^{*}=0,\ldots ,\lambda_{m}^{*}=0$ and that $A_{A}x^{*}=b_{A}$, system (34) can be reduced to the following one:

(43) $$\Phi \left(x,\lambda \right)=\left(\begin{array}{c} Qx+c+A_{A}^{T}\lambda_{A}\\ A_{A}x-b_{A} \end{array}\right)=0,$$

which can be written as:

(44) $$\left(\begin{array}{cc} Q & A_{A}^{T}\\ A_{A} & O_{p\times p} \end{array}\right)\;\left(\begin{array}{c} x\\ \lambda_{A} \end{array}\right)=\left(\begin{array}{c} -c\\ b_{A} \end{array}\right).$$

Under the assumptions LICQ and MSOSC, the following matrix is invertible,

$$\left(\begin{array}{cc} Q & A_{A}^{T}\\ A_{A} & O_{p\times p} \end{array}\right)$$

and system (44) yields the formula for the solution $\left(x^{*},\lambda^{*}\right)$:

(45) $$\left(\begin{array}{c} x^{*}\\ \lambda_{A}^{*} \end{array}\right)={\left[\begin{array}{cc} Q & A_{A}^{T}\\ A_{A} & O_{p\times p} \end{array}\right]}^{-1}\;\left(\begin{array}{c} -c\\ b_{A} \end{array}\right).$$

**Remark 3.**  *System (41) reduces to system (43) by removing equations $\Lambda_{N}A_{N}h^{1}$, corresponding to the nonactive constraints. Similarly, Equation (42) reduces to (45).*

**Remark 4.**  We note that solutions of QP problems have the following specific property: if $x^{*}$ is a solution of the QP problem and $h^{T}L_{xx}^{\prime \prime }\left(x,\lambda \right)h=0$ for the vector h∈KerA, then the points $x=x^{*}+th$ are also solutions of the QP problem.

#### 5.1.3. Examples

In this section, we illustrate the two described approaches with examples. Namely, we consider the construction of system (41) required for the first approach. Then we illustrate using the exact formula (45) derived in the second approach.

**Example 2.**  *Consider the problem:*

(46) $$\begin{array}{cc} \underset{x}{\mathrm{minimize}} & \frac{1}{2}x_{2}^{2}-x_{1}\\ \mathrm{subject}\;\mathrm{to} & x_{1}\leq 0,\\ \; & x_{2}\leq 1. \end{array}$$

The matrix A in this example is $A=\left(\begin{array}{cc} 1 & 0\\ 0 & 1 \end{array}\right)$ and $b=\left(\begin{array}{c} 0\\ 1 \end{array}\right).$ The solution to this problem is the point $\left(x_{1}^{*},x_{2}^{*}\right)=\left(0,0\right)$ with $\lambda_{1}^{*}=1$ and $\lambda_{2}^{*}=0$. Hence, $I\left(x^{*}\right)=\left\{1\right\}$, $A_{A}=\left(1,0\right)$, and $b_{A}=0$. Moreover,

$$Q=\left(\begin{array}{cc} 0 & 0\\ 0 & 1 \end{array}\right)\;and\;c=\left(\begin{array}{c} -1\\ 0 \end{array}\right).$$

*By choosing $h^{1}={\left(0,1\right)}^{T}$ and $h^{2}={\left(1,0\right)}^{T}$, the system (41) reduces to the linear system:*

$$\Psi \left(x,\lambda \right)=\left(\begin{array}{c} Qx+c+A^{T}\lambda \\ A_{A}x-b_{A}\\ \Lambda_{N}A_{N}h^{1} \end{array}\right)=\left(\begin{array}{c} \lambda_{1}-1\\ x_{2}+\lambda_{2}\\ x_{1}\\ \lambda_{2} \end{array}\right).$$

Solving the system Ψ(x,λ)=0 yields $\left(x_{1}^{*},x_{2}^{*},\lambda_{1}^{*},\lambda_{2}^{*}\right)=\left(0,0,1,0\right)$, as claimed.

Now, let us illustrate the second approach. Specifically, using the formula (45) for the solution of problem (46) with $\lambda_{A}=\lambda_{1}$, we obtain:

$$\left(\begin{array}{c} x_{1}^{*}\\ x_{2}^{*}\\ \lambda_{1}^{*} \end{array}\right)={\left[\begin{array}{ccc} 0 & 0 & 1\\ 0 & 1 & 0\\ 1 & 0 & 0 \end{array}\right]}^{-1}\;\left(\begin{array}{c} 1\\ 0\\ 0 \end{array}\right)=\left(\begin{array}{c} 0\\ 0\\ 1 \end{array}\right),$$

as claimed.

**Example 3.**  *Consider the problem:*

(47) $$\begin{array}{cc} \underset{x}{\mathrm{minimize}} & x_{1}^{2}+\frac{1}{2}x_{2}^{2}+x_{1}x_{2}\\ \mathrm{subject}\;\mathrm{to} & -x_{1}\leq 1,\\ \; & x_{2}\leq 1. \end{array}$$

The solution to this problem is the point $\left(x_{1}^{*},x_{2}^{*}\right)=\left(0,0\right)$ with $\lambda_{1}^{*}=0$ and $\lambda_{2}^{*}=0$. Hence, $I(x^{*})=\emptyset$. Moreover,

$$Q=\left(\begin{array}{cc} 2 & 1\\ 1 & 1 \end{array}\right)\;and\;c=\left(\begin{array}{c} 0\\ 0 \end{array}\right).$$

*By choosing $h^{1}={\left(1,1\right)}^{T}$ and $h^{2}={\left(0,0\right)}^{T}$, the system (41) reduces to the following linear system for problem (47):*

$$\Psi \left(x,\lambda \right)=\left(\begin{array}{c} 2x_{1}+x_{2}-\lambda_{1}\\ x_{1}+x_{2}+\lambda_{2}\\ -\lambda_{1}\\ \lambda_{2} \end{array}\right).$$

Solving Ψ(x,λ)=0 yields $\left(x_{1}^{*},x_{2}^{*},\lambda_{1}^{*},\lambda_{2}^{*}\right)=\left(0,0,0,0\right)$, as claimed.

To illustrate the second approach, we rewrite the exact formula (45) for the solution of problem (47) in the form:

(48) $$\left(\begin{array}{c} x_{1}^{*}\\ x_{2}^{*} \end{array}\right)={\left[\begin{array}{cc} 2 & 1\\ 1 & 1 \end{array}\right]}^{-1}\;\left(\begin{array}{c} 0\\ 0 \end{array}\right)=\left(\begin{array}{c} 0\\ 0 \end{array}\right),$$

as claimed.

#### 5.1.4. Third Approach to Solving the QP Problem

In this subsection, we present another approach to solving the QP problem. A formula that we obtain for the solution of the QP problem is also based on the construction of the 2-factor-operator.

First, we replace the inequality constraints in the QP problem with equality constraints of the form:

$$Ax-b+y^{2}=0,$$

where $y^{2}={\left(y_{1}^{2},\ldots ,y_{m}^{2}\right)}^{T}$. We then define the Lagrangian as follows:

(49) $$\tilde{L}\left(x,y,\lambda \right)=\frac{1}{2}x^{T}Qx+c^{T}x+\sum_{i=1}^{m}\lambda_{i}\left(a_{i}x-b_{i}+y_{i}^{2}\right).$$

Introduce the notation:

$$\Lambda =\mathrm{diag}{\left(\lambda_{j}\right)}_{j=1}^{m},\;Y=\mathrm{diag}{\left(y_{j}\right)}_{j=1}^{m},\;e={\left(1,1,\ldots ,1\right)}^{T}.$$

Then the point $\left(x^{*},y^{*},\lambda^{*}\right)$ is a solution of the following system:

(50) $$\tilde{\Phi }\left(x,y,\lambda \right)=\left(\begin{array}{c} Qx+c+A^{T}\lambda \\ 2\Lambda Ye\\ Ax-b+y^{2} \end{array}\right)=0.$$

The Jacobian matrix of the system (50) is given by:

$${\tilde{\Phi }}^{\prime }\left(x,y,\lambda \right)=\left(\begin{array}{ccc} Q & O_{n\times m} & A^{T}\\ O_{m\times n} & 2\Lambda & 2Y\\ A & 2Y & O \end{array}\right).$$

Then with $h={\left(h_{1},h_{2},h_{3}\right)}^{T},\;h_{1}\in R^{n},\;h_{2},h_{3}\in R^{m}$, we obtain

$${\tilde{\Phi }}^{\prime }\left(x,y,\lambda \right)h=\left(\begin{array}{c} Qh_{1}+A^{T}h_{3}\\ 2\Lambda h_{2}+2Yh_{3}\\ Ah_{1}+2Yh_{2} \end{array}\right).$$

Assuming that LICQ and MSOSC hold, matrix ${\tilde{\Phi }}^{\prime }\left(x^{*},y^{*},\lambda^{*}\right)$ is singular if and only if the strict complementarity condition does not hold. In other words, the set of indices of the weakly active constraints,

$$I_{0}\left(x^{*}\right)=\left\{j=1,\ldots ,m|\lambda_{j}^{*}=0,\;y_{j}^{*}=0\right\},$$

is not empty.

Let $P_{1}$ be the matrix of the orthoprojector onto ImΦ˜′(x*,y*,λ*), and $P_{2}$ be the matrix of the orthoprojector onto ImΦ˜′(x*,y*,λ*)⊥. Note that $P_{1}$ will be a projector onto the linear part of the mapping Φ, while $P_{2}$ will be a projector onto the quadratic part of Φ.

Introduce vector $\hat{h}=\left({\hat{h}}_{1},{\hat{h}}_{2},{\hat{h}}_{3}\right)$ such that $P_{2}{\tilde{\Phi }}^{\prime }\left(x^{*},y^{*},\lambda^{*}\right)\hat{h}=0$. Then,

$$\left\{\begin{array}{cc} 2\Lambda {\hat{h}}_{2}+2Y{\hat{h}}_{3} & =0\\ A{\hat{h}}_{1}+2Y{\hat{h}}_{2} & =0 \end{array}\right.$$

or

$${\left({\hat{h}}_{2}\right)}_{i}=\left\{\begin{array}{cc} k, & k\in R\setminus \left\{0\right\},\mathrm{when}y_{i}^{*}=0,\mathrm{and}\lambda_{i}^{*}=0,\\ 0, & \mathrm{when}y_{i}^{*}=0,\lambda_{i}^{*}\neq 0,\\ w, & w\in R\setminus \left\{0\right\},\mathrm{when}y_{i}^{*}\neq 0,\lambda_{i}^{*}=0, \end{array}\right.$$

$${\left({\hat{h}}_{3}\right)}_{i}=\left\{\begin{array}{cc} t, & t\in R\setminus \left\{0\right\},\mathrm{when}y_{i}^{*}=0,\mathrm{and}\lambda_{i}^{*}=0,\\ 0, & \mathrm{when}y_{i}^{*}\neq 0,\lambda_{i}^{*}=0,\\ r, & r\in R\setminus \left\{0\right\},\mathrm{when}y_{i}^{*}=0,\lambda_{i}^{*}\neq 0, \end{array}\right.$$

and ${\hat{h}}_{1}$ is defined by:

(51) $$A{\hat{h}}_{1}+2Y{\hat{h}}_{2}=0.$$

Observe that $P_{2}{\tilde{\Phi }}^{\prime }\left(x^{*},y^{*},\lambda^{*}\right)\hat{h}=0$, i.e., $\hat{h}\in \mathrm{Ker}P_{2}{\tilde{\Phi }}^{\prime }\left(x^{*},y^{*},\lambda^{*}\right)$.

Define *H* as a diagonal matrix with elements in the rows corresponding to the components of the vector ${\hat{h}}_{2}$, and *K* as a diagonal matrix with elements of the vector ${\hat{h}}_{3}$, so that:

$$H=\mathrm{diag}\left({\left({\hat{h}}_{2}\right)}_{i}\right),\;K=\mathrm{diag}\left({\left({\hat{h}}_{3}\right)}_{i}\right),\;i=1,\ldots ,m.$$

Then:

$${\tilde{\Phi }}^{\prime \prime }\left(x,y,\lambda \right)\hat{h}=\left(\begin{array}{ccc} O & O_{n\times m} & O\\ O_{m\times n} & 2K & 2H\\ O_{m\times n} & 2H & O \end{array}\right).$$

The 2-factor-operator for the mapping $\tilde{\Phi }$ is defined as:

$$\tilde{\Psi }\left(x,y,\lambda \right)=P_{1}\tilde{\Phi }\left(x,y,\lambda \right)+P_{2}{\tilde{\Phi }}^{\prime }\left(x,y,\lambda \right)\hat{h}$$

or

$$\tilde{\Psi }\left(x,y,\lambda \right)=\left(\begin{array}{c} Qx+c+A^{T}\lambda \\ 2\Lambda {\hat{h}}_{2}+2Y{\hat{h}}_{3}\\ A{\hat{h}}_{1}+2Y{\hat{h}}_{2} \end{array}\right).$$

We choose a vector ${\hat{h}}_{1}$ according to (51) so that matrix:

$$\tilde{\Psi }\left(x^{*},y^{*},\lambda^{*}\right)=\left(\begin{array}{ccc} Q & O_{n\times m} & A^{T}\\ O_{m\times n} & 2K & 2H\\ O_{m\times n} & 2H & O_{n\times m} \end{array}\right)$$

is nonsingular. Then $\left(x^{*},y^{*},\lambda^{*}\right)$ can be determined using the following formula:

$$\left(\begin{array}{c} x^{*}\\ y^{*}\\ \lambda^{*} \end{array}\right)={\left[\begin{array}{ccc} Q & O_{n\times m} & A^{T}\\ O_{m\times n} & 2K & 2H\\ O_{m\times n} & 2H & O_{m\times m} \end{array}\right]}^{-1}\;\left(\begin{array}{c} -c\\ O_{m}\\ -A{\hat{h}}_{1} \end{array}\right).$$

### 5.2. Identification of the Active Constraints

In this section, we address the issue of identifying the active constraints and propose strategies for numerically identifying the set of active constraints $I(x^{*})$.

We begin by considering the mapping $h\left(z\right):R^{n}\to R^{n}$, where $h\in C^{2}\left(R^{n}\right)$. We can also represent *h* as an *n*-vector of functions $h_{1}$, *…*, $h_{n}$, such that $h\left(z\right)={\left(h_{1}\left(z\right),\cdots ,h_{n}\left(z\right)\right)}^{T}$.

**Theorem 8.**  Let $h\in C^{2}\left(R^{n}\to R^{n}\right)$ be 2-regular at the point $z^{*}$, and let $N_{\varepsilon }\left(z^{*}\right)$ be a sufficiently small neighborhood of $z^{*}$ in $R^{n}$. Assume that there exists a function $\eta \left(z\right):N_{\varepsilon }\left(z^{*}\right)\to R$ such that $\eta (z^{*})=0$ and for all $z\in N_{\varepsilon }\left(z^{*}\right)$, we have:

(52) $$c_{1}∥z-z^{*}{∥}^{2}\leq \eta \left(z\right)\leq c_{2}∥z-z^{*}∥,$$

*where $c_{1},c_{2}>0$ are independent constants.*

Then there exists a sufficiently small δ such that 0<δ<ε, and for any 1≤i≤n and any point $z\in N_{\delta }\left(z^{*}\right)$, the following holds:

- Either $|h_{i}{\left(z\right)|>\eta \left(z\right)}^{1/3}$, which implies that $h_{i}\left(z^{*}\right)\neq 0,$
- Or $|h_{i}{\left(z\right)|<\eta \left(z\right)}^{1/3}$, which implies that $h_{i}\left(z^{*}\right)=0.$

**Proof.**  The proof is similar to the one in [5]. □

Let:

$$\varrho \left(x\right)=\min_{i=1,\ldots ,m}d\left(g_{i}^{\prime }\left(x\right),\mathrm{span}(g_{1}^{\prime }\left(x\right),\ldots ,g_{i-1}^{\prime }\left(x\right),g_{i+1}^{\prime }\left(x\right),\ldots ,g_{m}^{\prime }\left(x\right)\right),$$

where d(a,S) denotes the distance between a vector *a* and a set *S*. It turns out that if we take $\eta \left(x\right)=\max\left\{{∥g\left(x\right)∥}^{1/2},\varrho \left(x\right)\right\}$, and *g* is 2-regular at $x^{*}$, then inequality (52) holds with z=x.

Theorem 8 can be used for the numerical determination of the set of active constraints $I(x^{*})$ in the QP problem. To apply Theorem 8, we need to define a function η(·) that satisfies the conditions of the theorem. Recall that for QP problem (3), we denote the Lagrange function defined in (32) by L(x,λ).

Under the assumptions of LICQ and MSOSC, the following holds for $x\in N_{\varepsilon }\left(x^{*}\right)$ and $\lambda \in N_{\varepsilon }\left(\lambda^{*}\right)$:

$$c_{1}∥x-x^{*}∥\leq ∥L_{x}^{\;\prime }\left(x,\lambda \right)∥+\sum_{i=1}^{m}|\min\left\{\lambda_{i},b_{i}-a_{i}x\right\}|\leq c_{2}(∥x-x^{*}∥+∥\lambda -\lambda^{*}∥),$$

where ε>0 is sufficiently small (see, for example, [18]). Hence, the required function η(x,λ) can be defined by:

$$\eta \left(x,\lambda \right)=∥L_{x}^{\;\prime }\left(x,\lambda \right)∥+\sum_{i=1}^{m}\left|\min\left\{\lambda_{i},b_{i}-a_{i}x\right\}\right|.$$

Then, according to Theorem 8, for every i=1,…,m, if:

$$|b_{i}-a_{i}{x|\leq \eta \left(x,\lambda \right)}^{1/2},$$

then it follows that $i\in I(x^{*})$.

Moreover, if we introduce the function:

$$\tilde{\eta }\left(x,\lambda \right)=∥{\left(L_{A}\right)}_{x}^{\prime }\left(x,\lambda \right)∥+\sum_{i=1}^{m}\left|a_{i}x-b_{i}\right|,$$

where: $L_{A}\left(x,\lambda \right)=\frac{1}{2}x^{T}Qx+c^{T}x+\sum_{i=1}^{m}\lambda_{i}\left(a_{i}x-b_{i}\right)$, then $\tilde{\eta }\left(x,\lambda \right)$ satisfies the estimate:

$$c_{1}∥\left(x,\lambda \right)-\left(x^{*},\lambda^{*}\right)∥\leq \tilde{\eta }\left(x,\lambda \right)\leq c_{2}∥\left(x,\lambda \right)-\left(x^{*},\lambda^{*}\right)∥$$

for $\left(x,\lambda \right)\in N_{\varepsilon }\left(x^{*},\lambda^{*}\right)$, where ε>0 is a sufficiently small number.

Then, for any $i\in I(x^{*})$, if:

$$|\lambda_{i}|\leq \tilde{\eta }{\left(x,\lambda \right)}^{1/2},$$

then $i\in I_{0}\left(x^{*}\right)=I\left(x^{*}\right)\setminus I_{+}\left(x^{*}\right)$. Here, $I_{0}\left(x^{*}\right)$ represents the set of constraints that are weakly active, i.e, for which the associated multipliers are equal to zero, while $I_{+}\left(x^{*}\right)$ denotes the set of constraints that are strongly active at the point $x^{*}$, i.e., the associated Lagrange multipliers are positive.

### 5.3. General Case

Consider the Lagrange function in the form:

$$L\left(x,\lambda_{0},\lambda \right)=\lambda_{0}\left(\frac{1}{2}x^{T}Qx+c^{T}x\right)+\sum_{i=1}^{m}\lambda_{i}\left(a_{i}x-b_{i}\right).$$

In this case, if $x^{*}$ is a solution of problem (3), then there exist multipliers $\lambda_{0}^{*}$ and $\lambda^{*}$, not all zeros, such that $\lambda_{i}^{*}\geq 0$, $Ax^{*}\leq b$, and the point $\left(x^{*},\lambda_{0}^{*},\lambda^{*}\right)$ is a solution of the following system:

(53) $$\Phi_{0}\left(x,\lambda_{0},\lambda \right)=\left(\begin{array}{c} \lambda_{0}\left(Qx+c\right)+A^{T}\lambda \\ \Lambda (Ax-b)\\ \lambda_{0}+\sum_{i=1}^{m}\lambda_{i}-1 \end{array}\right)=0,\;\Lambda =\mathrm{diag}{\left(\lambda_{i}\right)}_{i=1,\ldots ,m}.$$

Introduce the notation:

(54) $$\xi \left(x,\lambda_{0},\lambda \right)=∥\lambda_{0}\left(Qx+c\right)+\sum_{i=1}^{m}\lambda_{i}a_{i}^{T}∥+\sum_{i=1}^{m}\left|\lambda_{i}\left(a_{i}x-b_{i}\right)\right|+\lambda_{0}+\sum_{i=1}^{m}\lambda_{i}-1.$$

We are making the following assumption for the rest of the section.

**Assumption A1.**  Assume that there exists $C_{1}>0$ and a sufficiently small ε>0 such that for any $\left(x,\lambda_{0},\lambda \right)\in N_{\varepsilon }\left(x^{*},\lambda_{0}^{*},\lambda^{*}\right)$, the following holds:

$$\xi \left(x,\lambda_{0},\lambda \right)\geq C_{1}{∥x-x^{*}∥}^{2}.$$

**Remark 5.**  It is easy to see that for any $\left(x,\lambda_{0},\lambda \right)\in N_{\varepsilon }\left(x^{*},\lambda_{0}^{*},\lambda^{*}\right)$,

$$\xi \left(x,\lambda_{0},\lambda \right)\leq C_{2}∥\left(x,\lambda_{0},\lambda \right)-\left(x^{*},\lambda_{0}^{*},\lambda^{*}\right)∥,$$

*where $C_{2}$ is an independent constant.*

As follows from Assumption 1 and Theorem 8, for those indices i=1,…,m that satisfy the inequalty:

$$|a_{i}x-b_{i}|<\xi {\left(x,\lambda_{0},\lambda \right)}^{1/4}$$

we make a conclusion that $i\in I(x^{*})$.

We can illustrate Assumption 1 with the following examples, where Assumption 1 holds.

**Example 4.**  *This example illustrates a choice of the function ξ in a more general setting.*

Consider mapping *F* defined by either:

$$F\left(x,\lambda \right)=\left(\begin{array}{c} x^{2}-\lambda^{2}\\ x\lambda \end{array}\right)$$

or

$$F\left(x,\lambda \right)=\left(\begin{array}{c} x-\lambda \\ x\lambda \end{array}\right).$$

In each of the two cases, $\left(x^{*},\lambda^{*}\right)=\left(0,0\right)$.

Introduce function ξ defined as:

ξ(x,λ)=∥F(x,λ)∥.

It follows that for any $\left(x,\lambda \right)\in N_{\varepsilon }\left(x^{*},\lambda^{*}\right)$, the inequality:

$$\xi \left(x,\lambda \right)\geq {C∥x-0∥}^{2}$$

holds, where *C* is an independent constant.

**Example 5.**  *Consider the problem:*

(55) $$\begin{array}{cc} \underset{x}{\mathrm{minimize}} & x_{2}^{2}-x_{1}\\ \mathrm{subject}\;\mathrm{to} & 2x_{1}\leq 0,\\ \; & x_{1}\leq 0. \end{array}\;\;.$$

The solution to this problem is the point $\left(x_{1}^{*},x_{2}^{*}\right)=\left(0,0\right)$, so $I\left(x^{*}\right)=\left\{1,2\right\}$. Moreover, the system (53) in this example is given by:

(56) $$\Phi_{0}\left(x,\lambda_{0},\lambda \right)=\left(\begin{array}{c} -\lambda_{0}+\lambda_{1}+\lambda_{2}\\ 2\lambda_{0}x_{2}\\ 2\lambda_{1}x_{1}\\ \lambda_{2}x_{1}\\ \lambda_{0}+\lambda_{1}+\lambda_{2}-1 \end{array}\right)=0.$$

We also introduce the function $\xi (x,\lambda_{0},\lambda )$, which can be defined using Equation (54), but in this case, we define it as $\xi \left(x,\lambda_{0},\lambda \right)=\left|\Phi_{0}\left(x,\lambda_{0},\lambda \right)\right|$.

Under Assumption 1, we use the function ξ to determine the set $I(x^{*})$. We also take into account the fact that the constraints in the problem are linear and the rank of the matrix

$$\left(\begin{array}{cc} 2 & 0\\ 1 & 0 \end{array}\right)$$

is 1. This implies that the constraints are linearly dependent. Consequently, we can eliminate, for instance, the second constraint from problem (55) and simplify system (56) to the following one:

$$\Phi_{0}\left(x,\lambda_{0},\lambda \right)=\left(\begin{array}{c} -\lambda_{0}+\lambda_{1}\\ 2\lambda_{0}x_{2}\\ 2\lambda_{1}x_{1}\\ \lambda_{0}+\lambda_{1}-1 \end{array}\right)=0.$$

Now, by introducing:

$$P_{1}=\left(\begin{array}{cccc} 1 & 0 & 0 & 0\\ 0 & 0 & 0 & 0\\ 0 & 0 & 0 & 0\\ 0 & 0 & 0 & 1 \end{array}\right),\;P_{2}=\left(\begin{array}{cccc} 0 & 0 & 0 & 0\\ 0 & 1 & 0 & 0\\ 0 & 0 & 1 & 0\\ 0 & 0 & 0 & 0 \end{array}\right),\;\;\mathrm{and}\;\;h=\left(\begin{array}{c} 0\\ 0\\ 1\\ 1 \end{array}\right),$$

we construct the modified 2-factor-system:

$${\tilde{\Phi }}_{0}\left(x,\lambda_{0},\lambda \right)=P_{1}\Phi_{0}\left(x,\lambda_{0},\lambda \right)+P_{2}\Phi_{0}^{\prime }\left(x,\lambda_{0},\lambda \right)h=\left(\begin{array}{c} -\lambda_{0}+\lambda_{1}\\ 2x_{2}\\ 2x_{1}\\ \lambda_{0}+\lambda_{1}-1 \end{array}\right)=0.$$

This system implies that the solution is $x^{*}=0$.

Now we will demonstrate the application of the approach described in Section 5.1.2 to problem (55). By removing the first constraint, we obtain a regular QP problem with $A_{A}=\left(1,0\right)$. Additionally, in this example,

$$Q=\left(\begin{array}{cc} 0 & 0\\ 0 & 1 \end{array}\right),\;c=\left(\begin{array}{c} -1\\ 0 \end{array}\right).$$

Then application of Equation (45) derived in Section 5.1.2 yields:

$$\left(\begin{array}{c} x_{1}^{*}\\ x_{2}^{*}\\ \lambda^{*} \end{array}\right)={\left[\begin{array}{ccc} 0 & 0 & 1\\ 0 & 1 & 0\\ 1 & 0 & 0 \end{array}\right]}^{-1}\;\left(\begin{array}{c} 1\\ 0\\ 0 \end{array}\right)=\left(\begin{array}{c} 0\\ 0\\ 1 \end{array}\right),$$

as claimed.

There are various directions in which the approach proposed in this paper can be extended. The next example illustrates a degenerate QP problem, in which MSOSC does not hold at the solution. However, an approach proposed in this paper can be applied to find a solution to this problem. Moreover, the solution set is locally not unique.

There are various directions in which the approach proposed in this paper can be extended. The next example illustrates a degenerate QP problem in which MSOSC does not hold at the solution. However, the approach proposed in this paper can still be applied to find a solution to this problem. It is worth noting that the solution set in this case is locally not unique.

**Example 6.**  *Consider the problem:*

(57) $$\begin{array}{cc} \underset{x}{\mathrm{minimize}} & x_{1}x_{2}-x_{1}\\ \mathrm{subject}\;\mathrm{to} & x_{1}\leq 0. \end{array}\;\;.$$

The solution to this problem is the set of points $X^{*}=\left\{\left(0,x_{2}^{*}\right)|x_{2}^{*}\in R\right\}$. We observe that the objective function in this example is satisfied as an equality for any $x^{*}\in X^{*}$. Additionally, the system (53) for this example consists of one linear equation and three quadratic equations:

$$\Phi_{0}\left(x,\lambda_{0},\lambda \right)=\left(\begin{array}{c} \lambda_{0}x_{2}-\lambda_{0}+\lambda_{1}\\ \lambda_{0}x_{1}\\ \lambda_{1}x_{1}\\ \lambda_{0}+\lambda_{1}-1 \end{array}\right)=0.$$

Denote the projection of the point *x* onto the set $X^{*}$ by $P_{X^{*}}\left(x\right)$. Also, define the notation $\xi \left(x,\lambda_{0},\lambda \right)=∥\Phi_{0}\left(x,\lambda_{0},\lambda \right)∥$. For any point $\left(x,\lambda_{0},\lambda \right)\in N_{\varepsilon }\left(x^{*},\lambda_{0}^{*},\lambda^{*}\right)$, we have the inequality:

$$\xi \left(x,\lambda_{0},\lambda \right)\geq \alpha ∥x-P_{X^{*}}\left(x\right)∥,$$

where α>0 and ε>0 is sufficiently small.

Consider, for example, the point $x^{*}={\left(0,1\right)}^{T}$.

In problem (57), we replace the inequality $x_{1}\leq 0$ with the equation $x_{1}+y^{2}=0$, where y∈R. We then introduce the Lagrange function in the form of (49) as follows:

$$L\left(x,y,\lambda \right)=\left(x_{1}x_{2}-x_{1}\right)+\lambda \left(x_{1}+y^{2}\right).$$

If $\lambda^{*}$ is a Lagrange multiplier corresponding to the solution $\left(x_{1}^{*},x_{2}^{*},y^{*}\right)=\left(0,x_{2}^{*},0\right)$, then the point $\left(x_{1}^{*},x_{2}^{*},y^{*},\lambda^{*}\right)$ is a solution of the following system:

(58) $$\Phi \left(x_{1},x_{2},y,\lambda \right)=\left(\begin{array}{c} x_{2}-1+\lambda \\ x_{1}\\ 2\lambda y\\ x_{1}+y^{2} \end{array}\right)=0.$$

The Jacobian matrix of this system is given by:

$$\Phi^{\prime }\left(x_{1},x_{2},y,\lambda \right)=\left(\begin{array}{cccc} 0 & 1 & 0 & 1\\ 1 & 0 & 0 & 0\\ 0 & 0 & 2\lambda & 2y\\ 1 & 0 & 2y & 0 \end{array}\right).$$

This Jacobian matrix becomes singular at $\left(x_{1},x_{2},y,\lambda \right)=\left(0,x_{2},0,\lambda \right)$. To overcome this singularity, we can apply the approach described in the paper. Specifically, we notice that $\Phi^{\prime }\left(x_{1}^{*},x_{2}^{*},y^{*},\lambda^{*}\right)h=0$ for h=(0,1,1,−1). Moreover, the point $\left(x_{1}^{*},x_{2}^{*},y^{*},\lambda^{*}\right)=\left(0,1,0,0\right)$ is one of the solutions of the system defined in (58), corresponding to a solution of the QP problem (57).

Additionally,

$$\Phi^{\prime }\left(x_{1},x_{2},y,\lambda \right)\;\left(\begin{array}{c} 0\\ 1\\ 1\\ -1 \end{array}\right)=\left(\begin{array}{c} 0\\ 0\\ 2\lambda -2y\\ 2y \end{array}\right),\;\Phi^{\prime \prime }\left(x_{1},x_{2},y,\lambda \right)\;\left(\begin{array}{c} 0\\ 1\\ 1\\ -1 \end{array}\right)=\left(\begin{array}{cccc} 0 & 0 & 0 & 0\\ 0 & 0 & 0 & 0\\ 0 & 0 & -2 & 2\\ 0 & 0 & 2 & 0 \end{array}\right).$$

The 2-factor-operator of $\Phi (x_{1}^{*},x_{2}^{*},y^{*},\lambda^{*})$ with respect to the vector h=(0,1,1,−1), which is defined similarly to the operator in Equation (11), is given by:

$$\Phi^{\prime }\left(x_{1}^{*},x_{2}^{*},y^{*},\lambda^{*}\right)+\Phi^{\prime \prime }\left(x_{1}^{*},x_{2}^{*},y^{*},\lambda^{*}\right)h=\left(\begin{array}{cccc} 0 & 1 & 0 & 1\\ 1 & 0 & 0 & 0\\ 0 & 0 & -2 & 2\\ 1 & 0 & 2 & 0 \end{array}\right).$$

Note that the 2-factor-operator is nonsingular and the system:

$$\Phi \left(x_{1},x_{2},y,\lambda \right)+\Phi^{\prime }\left(x_{1},x_{2},y,\lambda \right)h=\left(\begin{array}{c} x_{2}-1+\lambda \\ x_{1}\\ 2\lambda y+2\lambda -2y\\ x_{1}+y^{2}+2y \end{array}\right)=0$$

has the point $\left(x_{1}^{*},x_{2}^{*},y^{*},\lambda^{*}\right)=\left(0,1,0,0\right)$ as its regular solution.

## 6. Conclusions

The paper focused on applying the *p*-regularity theory to nonlinear equations with quadratic mappings and quadratic programming (QP) problems. The first part of the paper used the special structure of the nonlinear equation and the construction of a 2-factor operator to derive a formula for the solution of the equation. In the second part, the QP problem was reduced to a system of linear equations using a 2-factor-operator. The solution of the system is a local minimizer of the QP problem with a corresponding Lagrange multiplier. The formula for the solution of the linear system was given. The paper also described a procedure for identifying the active constraints, which was used in constructing the linear system.

The paper primarily focuses on the case where the matrix $F^{\prime }\left(x^{*}\right)$ is degenerate at the solution of the nonlinear equation F(x)=0. However, the matrix $F^{\prime }\left(x^{*}\right)$ does not need to be degenerate. While we do not explicitly address the identification of degeneracy at a solution point, it is possible to determine the degeneracy of the matrix $F^{\prime }\left(x^{*}\right)$ by examining the behavior of the mapping *F* in a small neighborhood of the solution $x^{*}$. Specifically, a function $\nu_{p}\left(x\right)$ can be defined, such that:

$$c_{1}∥x-x^{*}{∥}^{p}\leq \nu_{p}\left(x\right)\leq c_{2}∥x-x^{*}∥$$

for some natural number *p* and constants $c_{1}$ and $c_{2}$. Based on the conclusion about the degeneracy of the matrix $F^{\prime }\left(x^{*}\right)$, an appropriate method can be chosen to solve the system of equations F(x)=0, as stated in the following theorem.

**Theorem 9.**  Let $F\in C^{p}\left(R^{n},R^{n}\right)$ be such that $F(x^{*})=0$, and let there exist $x\in N_{\varepsilon }\left(x^{*}\right)$, where ε>0 is sufficiently small. Then we have the following two cases:

- *In the first case, for all i∈{1,2,…,n}, we have:*
$$d\left(f_{i}^{\prime }\left(x\right),\mathrm{span}\left(f_{1}^{\prime }\left(x\right),\ldots ,f_{i-1}^{\prime }\left(x\right),f_{i+1}^{\prime }\left(x\right),\ldots ,f_{m}^{\prime }\left(x\right)\right)\right)>\nu_{p}{\left(x\right)}^{1/p+1}.$$
In this case, $\det\;F^{\prime }\left(x^{*}\right)\neq 0$, indicating that F is not degenerate at $x^{*}$.
- *In the second case, there exists an index i∈{1,2,…,n} such that:*
$$d\left(f_{i}^{\prime }\left(x\right),\mathrm{span}\left(f_{1}^{\prime }\left(x\right),\ldots ,f_{i-1}^{\prime }\left(x\right),f_{i+1}^{\prime }\left(x\right),\ldots ,f_{m}^{\prime }\left(x\right)\right)\right)<\nu_{p}{\left(x\right)}^{1/p+1}.$$
In this case, $\det\;F^{\prime }\left(x^{*}\right)=0$, indicating that F is degenerate at $x^{*}$.

Certainly, the construction of the function $\nu_{p}\left(x\right)$ is an important consideration. One approach to constructing such a function is provided in the following lemma, specifically for the case of p=2.

**Lemma 3.**  Let $F\in C^{2}\left(R^{n},R^{n}\right)$ and assume that either ${\left(F^{\prime }\left(x^{*}\right)\right)}^{-1}$ exists, or for any $h\in KerF^{\prime }\left(x^{*}\right)$, there exists ${\left(F^{\prime \prime }\left(x^{*}\right)h\right)}^{-1}$ with ∥h∥=1. Then, there exists a sufficiently small ε>0 such that the following inequality holds for all $x\in N_{\varepsilon }\left(x^{*}\right)$:

$$∥F\left(x\right)∥\geq C∥x-x^{*}{∥}^{2},$$

*where C is a positive constant.*

Based on this lemma, one can choose the function $\nu_{2}\left(x\right)=∥F\left(x\right)∥$.

It is worth noting that the proposed approach also covers the case where the system of equations consists of both linear and quadratic equations. Moreover, the approach can be extended to solve multilinear equations with polynomials of degree *p*, given by the equation:

$$F\left(x\right)=Q_{p}{\left[x\right]}^{p}+Q_{p-1}{\left[x\right]}^{p-1}+\ldots +Q_{1}\left[x\right]+Q_{0}=0,$$

where $Q_{k}{\left[x\right]}^{k}$ is *k*-multilinear mapping for k=1,…,p. Additionally, polynomial programming problems can be formulated as follows:

$$\begin{array}{cc} \mathrm{minimize} & f(x)\\ \mathrm{subject}\;\mathrm{to} & f_{i}\left(x\right)\leq 0,\;i=1,\ldots ,m, \end{array}$$

where $f_{i}\left(x\right)$ are polynomial mappings.

There are various possible directions for future research work, based on the results obtained in this paper. While the focus of the current work was on obtaining exact formulas for the solutions of nonlinear equations with quadratic mappings and quadratic programming problems, it would be interesting to generalize the proposed approaches to other classes of problems, including systems of equations with both linear and quadratic mappings. Another direction would be an extension of presented methods to polynomial equations and polynomial programming problems. Another direction of future research could be focusing on numerical studies and the implementation of the methods described in the paper.

## Data Availability

No new data were created or analyzed in this study. Data sharing is not applicable to this article.
